# The Development of a Database for Herbal and Dietary Supplement Induced Liver Toxicity

**DOI:** 10.3390/ijms19102955

**Published:** 2018-09-28

**Authors:** Jieqiang Zhu, Ji-Eun Seo, Sanlong Wang, Kristin Ashby, Rodney Ballard, Dianke Yu, Baitang Ning, Rajiv Agarwal, Jürgen Borlak, Weida Tong, Minjun Chen

**Affiliations:** 1 Division of Bioinformatics and Biostatistics, National Center for Toxicological Research, U.S. Food and Drug Administration, Jefferson, AR 72079, USA; Jieqiang.Zhu@fda.hhs.gov (J.Z.); Jieun.Seo@fda.hhs.gov (J.-E.S.); wangsanlong@nifdc.org.cn (S.W.); kristin.mceuen@fda.hhs.gov (K.A.); rodney.ballard@fda.hhs.gov (R.B.); dianke.yu@fda.hhs.gov (D.Y.); baitang.ning@fda.hhs.gov (B.N.); 2 National Center for Safety Evaluation of Drugs, National Institutes for Food and Drug Control, China’s State Food and Drug Administration, Beijing 100176, China; 3 Center for Drug Evaluation and Research, U.S. Food and Drug Administration, Silver Spring, MD 20993, USA; Rajiv.Agarwal@fda.hhs.gov; 4 Center of Pharmacology and Toxicology, Hannover Medical School, 30625 Hannover, Germany; Borlak.Juergen@mh-hannover.de

**Keywords:** herbal and dietary supplements, liver injury, hepatotoxicity, drug interaction, database

## Abstract

The growing use of herbal dietary supplements (HDS) in the United States provides compelling evidence for risk of herbal-induced liver injury (HILI). Information on HDS products was retrieved from MedlinePlus of the U.S. National Library of Medicine and the herbal monograph of the European Medicines Agency. The hepatotoxic potential of HDS was ascertained by considering published case reports. Other relevant data were collected from governmental documents, public databases, web sources, and the literature. We collected information for 296 unique HDS products. Evidence of hepatotoxicity was reported for 67, that is 1 in 5, of these HDS products. The database revealed an apparent gender preponderance with women representing 61% of HILI cases. Culprit hepatotoxic HDS were mostly used for weight control, followed by pain and inflammation, mental stress, and mood disorders. Commonly discussed mechanistic events associated with HILI are reactive metabolites and oxidative stress, mitochondrial injury, as well as inhibition of transporters. HDS–drug interactions, causing both synergistic and antagonizing effects of drugs, were also reported for certain HDS. The database contains information for nearly 300 commonly used HDS products to provide a single-entry point for better comprehension of their impact on public health.

## 1. Introduction

Herbal and dietary supplements (HDS) include a broad range of products, e.g., herbal products, vitamins, minerals, amino acids, and other supplements. Per the U.S. Dietary Supplement Health and Education Act of 1994, HDS are not classified as “drug” but as “food (or dietary supplements)” and therefore are not stringently regulated by the U.S. Food and Drug Administration (FDA) without requiring mandatory safety or toxicological evaluations, which are usually required for conventional drugs [1,2]. Since consumers hold the general belief that HDS are made of natural ingredients and therefore are safe [3], HDS are widely used for multiple purposes, such as improving general well-being, “boosting” the immune system, improving weight loss, building muscle, and as complementary or alternative medications for various diseases [4,5]. HDS are readily accessible over-the-counter in pharmacies, health stores, department stores, and on the internet. A nationally representative cross-sectional survey conducted from 2003 to 2006 suggested that 49% of the U.S. population and 70% of adults who are ≥70-years old use HDS [6], and the sale of HDS in the United States has steadily increased over the past decade [6,7].

Between 2004 and 2013, over 400 New Dietary Ingredient (NDI) applications were submitted to the FDA for approval as new botanicals [8]. Meanwhile, herbal products such as Usnic Acid, OxyELITE Pro, and Hydroxycut received warnings from the FDA for their hepatotoxicity [9]. Very recently, flavocoxid products (Limbrel) consisting of plant-derived flavonoids were withdrawn from the market due to reports of severe hepatotoxicity [10]. Indeed, an on-going prospective multicenter study conducted by Drug-Induced Liver Injury Network (DILIN) showed that, in the United States, the proportion of hepatotoxic events related to HDS increased from 7% to 20% over the course of the study period (2004–2013) [11,12]. Moreover, a report from the 2002 National Health Interview Survey pointed out that only 33% of HDS users disclose HDS use to their physicians [13]. These low HDS disclosure rates, especially among individuals with chronic medical conditions, and the growing use of HDS among the general population make regulatory agencies increasingly concerned about the potential toxic risks of HDS use, including herbal-induced liver injury (HILI) [14,15].

We previously developed the Liver Toxicity Knowledge Base (LTKB) to enhance the understanding of liver toxicity caused by conventional drugs [16,17,18,19,20,21,22,23,24,25,26,27,28]. Here, we consider liver toxicity caused by HDS and establish a reference database to facilitate HILI research and provide a resource for clinicians in decision-making when suspected HDS hepatotoxicity is encountered. Our database includes HDS hepatotoxicity mechanisms, HDS–drug interactions, and other relevant data for 296 commercial HDS, 67 of which were the culprit in one or more published liver injury cases and are therefore identified as hepatotoxic in our HDS database.

## 2. Results

**Figure 1:**
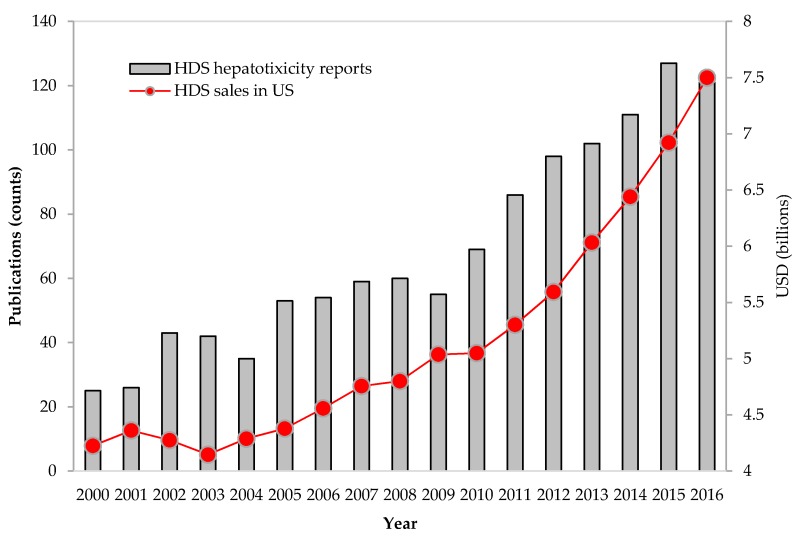
Increases in herbal dietary supplement (HDS) sales and in publications reporting HDS hepatotoxicity. Total sales for HDS have steadily increased in the U.S. market. With increasing HDS use, publications on liver injury due to HDS also have increased accordingly. The sources of HDS sales and publications are from the market reports in HerbalGram (http://abc.herbalgram.org/site/PageServer) and PubMed (accessed on 1 September 2017), respectively.

Publications related to herbal hepatotoxicity have increased about two times from 2006 to 2016 (Figure 1). Furthermore, the numbers of HILI-related publications vs. HDS sales in the United States appear to be proportional, thus suggesting that the increasing reports of HDS hepatotoxicity could be largely driven by the increased use of HDS.

### 2.1. HDS Use and Associated Hepatotoxicity

**Figure 2:**
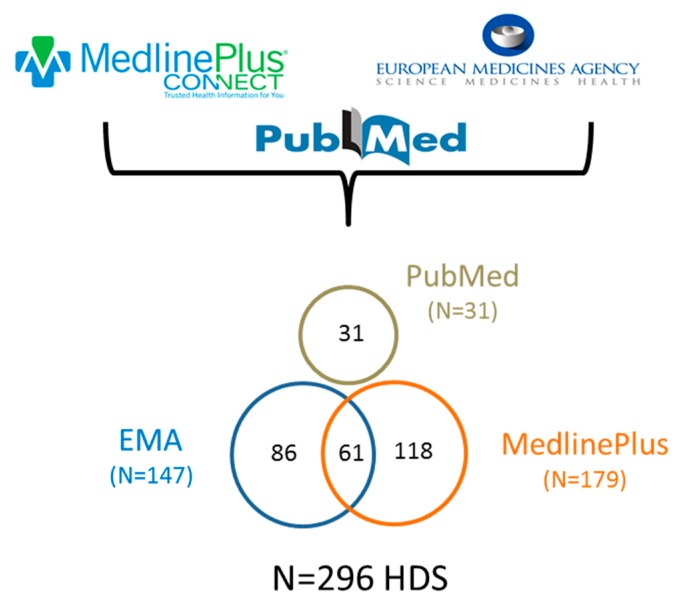
Data sources for the collection of 296 herbal and dietary supplements (HDS) evaluated for hepatotoxicity.

Certain HDS use could cause hepatotoxicity in humans. In our study, a total of 296 unique HDS were investigated, of which N = 147 HDS were from the European Medicines Agency (EMA), N = 179 HDS were from MedlinePlus, and N = 31 HDS were collected from PubMed (Figure 2). Of the 296 HDS, 61 were shared by two sources from EMA and MedlinePlus.

**Figure 3:**
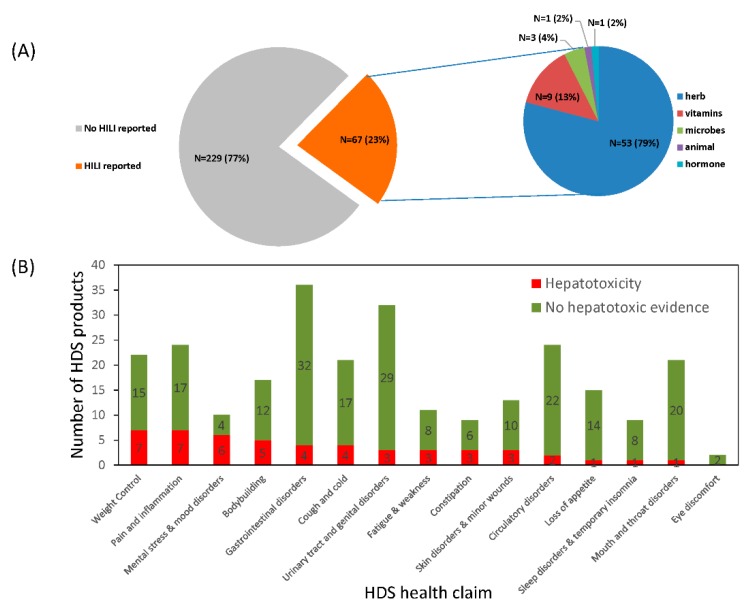
HDS use and associated hepatotoxicity. (**A**) The distribution of HDS hepatotoxicity. An HDS was evidenced as hepatotoxic when it was identified as the culprit cause of the injury in at least one qualified case report. (**B**) The distribution of human use of HDS with and without evidence of hepatotoxicity.

After searching for HDS hepatotoxicity case reports in the literature, 67 HDS (or 23% of the 296 HDS) were reported as the suspected culprit cause of liver injury for at least one qualified case with causality adjudicated and thus were categorized as potentially hepatotoxic (detailed in Table S1). Most hepatotoxic HDS were herbal products (N = 53, 79%), followed by vitamins (N = 9, 13%) and microbes (N = 3, 5%) as shown in Figure 3A. Of the 14 HDS health claim categories, weight loss, pain and inflammation, mental stress and mood disorders, and bodybuilding included the greatest number of HDS associated with hepatotoxic risk. HDS used for mouth and throat disorders and loss of appetite were seldom associated with hepatotoxicity (Figure 3B). In all categories except for the body building category, female HILI cases outnumbered males and overall represented 61% of the collected HILI hepatotoxicity cases.

### 2.2. Mechanism of HDS Hepatotoxicity

**Figure 4:**
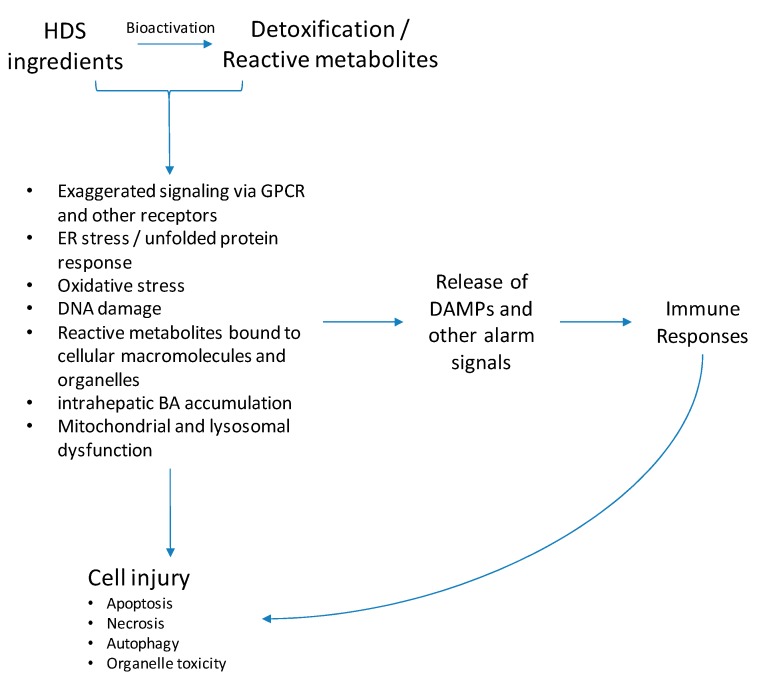
Potential mechanisms of HDS-induced liver injury. Most HDS hepatotoxicity develops through direct hepatocyte toxicity or by triggering an immune response. Toxic ingredients or their reactive metabolites cause direct hepatocyte toxicity through various mechanisms, such as oxidative stress, endoplasmic reticulum (ER) stress, DNA damage, toxic bile acids accumulation, and mitochondrial dysfunction, consequently leading to cell apoptosis or necrosis. Another important mechanism resulting in cell damage occurs when either toxic HDS ingredients, reactive metabolites, or danger damage-associated molecular patterns (DAMPs) released by stressed hepatocytes trigger an immune response. The exact mechanism leading to HDS-hepatotoxicity is still under investigation and other pathways may be involved.

The current understanding of the pathogenesis of HDS hepatotoxicity is limited partly due to the complicated mixture of ingredients in herbal products. Like conventional drugs, studies suggest that most HDS hepatotoxicity develops either through direct hepatocyte toxicity or by triggering an immune response [29,30] (Figure 4).

Many herbal ingredients are transformed into reactive metabolites via bioactivation process (Table S2). For example, monocrotaline is a pyrrolizine alkaloid that is likely metabolized by the hepatic CYP monooxygenase system into reactive intermediates, namely, monocrotaline pyrrole, which are toxic to cells [31,32]. These reactive species generated from HDS products have a high potential to bind with biomolecules, such as proteins, lipids, nucleic acids, and deplete antioxidant glutathione (GSH), resulting in the loss of organelle activities thereby inducing cellular stress responses.

**Figure 5:**
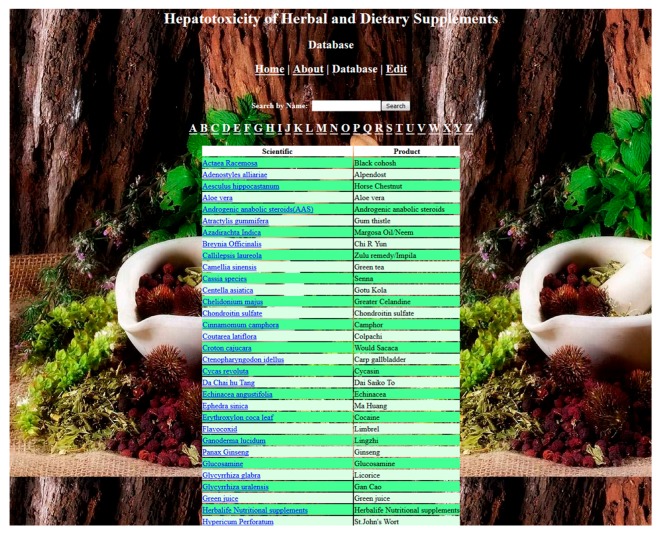
The screenshot of the Herbal and Dietary Supplements Hepatotoxicity Database (currently only for FDA users).

Toxic HDS ingredients and their reactive metabolites can initiate direct hepatocyte toxicity through various mechanisms, such as oxidative stress, endoplasmic reticulum (ER) stress, DNA damage, toxic bile acids accumulation, and mitochondrial dysfunction (Figure 5). The mitochondria play a central role in most of these processes, which can eventually result in massive mitochondrial injury, leading to mitochondrial permeability transition (MPT) and consequently cell apoptosis or necrosis. Oxidative stress is the most frequently reported mechanism of HDS-induced toxicity and is seen in the toxicity studies of kava [33], green tea extracts [34], usnic acid [35], greater celandine [36], chaparral [37], and black cohosh [38] (Table S3). Studies also found ER stress induced by chaparral [39,40]. In addition to mechanisms involved in direct hepatotoxicity, certain HDS ingredients also trigger protective cell mechanisms. For example, the induction of autophagy and c-Jun N-terminal kinase (JNK) activation, which prevent apoptosis and cell death, is seen in usnic-acid-induced toxicity [41].

Triggering an immune response is another important mechanism that results in cell damage (Figure 4). Although experimental studies of HDS-induced immunoallergic liver injury are scarce, a significant amount of clinical evidence implicates the immune response in hepatotoxicity induced by HDS such as ephedra, khat, and black cohosh [42,43,44]. Immunoallergic reaction symptoms (e.g., fever, rash, itching, eosinophilia), abnormal auto-antibodies (e.g., antinuclear antibody, antimitochondrial antibodies, smooth muscle antibodies), as well as immune cell infiltration (e.g., neutrophils, eosinophils) observed in liver biopsies were frequently reported.

### 2.3. HDS and Drug Interactions

HDS–drug interactions are of critical importance in clinical practice, but health-care professionals and patients are frequently unaware of the risks of hepatotoxicity arising from such interactions. When concomitantly given with conventional drugs, certain HDS have the potential to either induce or repress CYP enzymes and therefore change the ADME (i.e., absorption, distribution, metabolism, and excretion) profile of drugs in a way that could predispose some individuals to hepatotoxicity [45,46]. By inducing CYP enzymes, HDS can increase the amount of reactive metabolites produced from concomitant drugs. For example, Germander is a known cause of hepatotoxicity and can be bioactivated by CYP3A4 [47], and the pharmacological induction of the P450 enzymes by other drugs or alcohol could subsequently increase herbal toxic metabolites and associated toxicity [48,49]. The increased toxic metabolites may lead to cellular damage through diverse mechanisms such as oxidative stress, mitochondrial injury, glutathione depletion, DNA damage, and endoplasmic reticulum stress [18]. Furthermore, toxic metabolites can activate innate/adaptive immune response by releasing pro-inflammatory cytokines and/or inducing inflammation.

Within our database, certain hepatotoxic HDS induce or repress CYP enzymes activities. For example, St. John’s Wort is an inducer of CYP3A4 and can significantly increase hepatotoxicity of other agents through HDS–drug interaction [50,51]. Kavalactones, the active ingredients of kava, have been shown to be potent inhibitors of several CYP isozymes, such as CYP1A2, CYP2C9, and CYP2D6 [52].

## 3. Discussion

The rapid rise of HDS use is of concern for clinicians and regulatory agencies. Indeed, the estimated total sales of HDS have seen a tremendous increase in the U.S. consumer market over the past decade [7]. According to a survey from the Nutrition Business Journal, American consumers spent approximately $7.5 billion on HDS, a 7.7% year-over-year increase in 2016, and the sales in the past 10 years have grown by >50% [53]. Given the significant rise of HDS hepatotoxicity case reports [12], a reference database is needed to inform consumers and physicians of the hepatotoxicity potential associated with the use of HDS.

Here, we developed an HDS database as an independent component of the LTKB by aggregating HDS-hepatotoxicity-related data. Through this practice, we comprehensively investigated 296 commercial HDS commonly used in the U.S. and European markets and identified 67 HDS, or 1 in 5 HDS, reported to cause hepatotoxicity. This observation is significant considering that the sale of HDS in the United States has tremendously increased in recent years, which has driven the growing case reports of HDS hepatotoxicity [11,53]. 

Causality assessment is a significant challenge for HDS hepatotoxicity study due to the absence of specific diagnosis biomarkers. Some diseases, such as biliary diseases, viral infection, and alcoholic liver disease, could complicate the causality assessment process [54,55]. In practice, scoring instruments, such as Roussel Uclaf Causality Assessment Method (RUCAM) [56] and diagnosis based on expert opinion, were both applied [57], although diverse outcomes among these causality assessment scales were observed [58]. In our study, the culprit HDS in over 90% of cases was adjudicated for causality of observed hepatotoxicity using either expert justification or a scoring instrument such as RUCAM. The causality justification for the cases we collected relied on the assessment conducted by the original authors and was not re-evaluated due to the limited information available.

A total of 67 HDS products were identified as culprits in at least one qualified hepatoxic case and we therefore categorized these as potentially hepatotoxic. Among them were 16 culprits with accidental rechallenge in addition to 17 HDS products with case of fatal outcome. Note that, according to the reporting physician adjudication, only some of the HDS products were considered as likely proven while the majority are considered as “possible” or “probable”, and therefore, causality is suspected rather than proven.

Recently, the NIH LiverTox database introduced an approach to categorize drugs implicated in causing DILI into five categories from those with the strongest evidence with over 50 published cases (category A) to those without any published cases (category E) [59]. In future work, we plan to include a risk stratification approach to further categorize the risk of HDS-induced liver injury in terms of both the quality and quantity of published case reports. Applying the methodology to the HDS hepatotoxicity classification could provide a better indication of the public health impact related to a given HDS product.

The interactions between HDS and conventional drugs also impact public health and are often underestimated. HDS–drug interactions can change the pharmacokinetic profile of drugs, potentially increasing hepatotoxic risk in susceptible individuals. In addition, HDS might also induce or repress the activity of various CYP isozymes to mediate the hepatotoxicity caused by concomitant drugs. For these reasons, when administrated along with HDS and conventional drugs either for a medical need or cosmetic use, there exists the potential for increased hepatotoxic effects of the HDS as well as adverse reactions to the drugs. Accordingly, concurrent use of HDS with the specific drugs should be avoided or carefully monitored.

Many reports suggest that females were associated with a higher risk of hepatotoxicity [45,60,61]. A prospective study from US DILIN showed that HDS hepatotoxicity more frequently led to severe clinical outcomes, including liver transplantation and death, as compared to injury from conventional medications (13% vs. 3%; *p* < 0.05), and these HDS hepatotoxicity cases were predominantly observed in middle-aged women [12]. Another prospective nationwide hepatotoxicity study in South Korea reported that 84% patients with HDS-related hepatotoxicity were woman, significantly higher than those caused by conventional medications (63%, *p* = 0.002) [62]. Women frequently use HDS for weight reduction, beauty enhancing, anti-aging, and rejuvenating effects [7] and HDS users in United States are most often women over age 40 with a high level of education [61,63,64]. In our database, HDS hepatotoxicity cases were over-presented by women (approximately 61% cases); however, it is largely unknown whether this observation is due to a greater susceptibility in women or other as yet unknown factor such as disproportional use of HDS in women [20].

An ethnic difference was also observed in hepatotoxicity associated with HDS use. Southeast Asian, African, and Central American ethnic groups traditionally use HDS and therefore might be at a higher risk for HDS hepatotoxicity [65,66]. Prospective hepatotoxicity studies from Korea and Singapore support this by showing a higher prevalence of hepatotoxicity due to HDS (73% and 71%, respectively) [65,67] than those with lesser HDS use, as seen by incidence of hepatotoxicity from the United States and the European Union (9–16% and 6–16%, respectively) [67,68]. In the United States, 30% of Asian Americans, 30% of Hispanics, and 17% of African Americans are reported as regular HDS users [69]. Our database shows these minorities more often consume potentially hepatotoxic HDS, such as Aloe Vera, Chi R Yun, Ginseng, St. John’s Wort, Cascara, and Kombucha tea, increasing their risk for developing HDS hepatotoxicity.

Altogether, HDS are not as safe as the consumers generally believe. Rather, HDS are made of complex mixtures of natural products and many have various side effects and potential toxicity. The HDS hepatotoxicity database we developed will provide consumers with an up-to-date HDS hepatoxicity resource and support the FDA’s regulatory decision. Ongoing curation will ensure a current and quality database of HDS hepatotoxicity.

## 4. Materials and Methods

### 4.1. Compilation of the HDS List

To comprehensively survey hepatotoxicity associated with the use of commercial HDS, we combined the HDS list from two authoritative resources, i.e., MedlinePlus [70] and the European Union herbal monograph [71], and included additional HDS from PubMed (Figure 2). The U.S. National Library of Medicine established the MedlinePlus database by compiling U.S.-marketed HDS from different agencies, including the National Center for Complementary and Integrative Health, Natural Medicines Comprehensive Database, National Toxicology Program, National Cancer Institute, and Office of Dietary Supplements in NIH [70]. The European Union herbal monograph and list entries is a resource compiled by the Committee on Herbal Medicines Products of the European Medicines Agency (EMA) for assessing the safety and efficacy of herbal substances in the European market [71].

### 4.2. Determination of HDS Hepatotoxicity

HDS hepatotoxic potential was evaluated by considering case reports published in the literature, primarily retrieved by searching PubMed, Google Scholar, NIH LiverTox database [72], and the European Union herbal monograph [71]. In the literature search, we used the terms “dietary supplement, herbal, herbal dietary supplement (HDS), botanical, herbal medicine, herb-induced liver injury, hepatotoxicity, liver damage, liver injury, liver toxicity, drug interaction, and cytochrome P450 (CYP)”. Other keywords such as “African American, Hispanic American, Asian American, ethnic groups, minorities, female, weight loss, obesity, sport, athletes, bodybuilding, and health enhancement” were also used to search for high-risk populations. The search was conducted on 1 May 2018.

HDS hepatotoxicity cases were selected based on whether case reports included the following information: serum biochemistries and/or liver biopsy findings, justification of a single HDS as the culprit of injury, and detailed clinical descriptions such as gender, age, administration of HDS, course of injury development, and clinical outcomes. We focused on reports written in English. Most of the retrieved HDS hepatotoxicity case reports were observational studies published in peer-reviewed journals. All cases were manually reviewed by two independent investigators to determine whether a case met inclusion criteria, and consensus meetings were held for any potential disagreements between the two investigators. An HDS was categorized as hepatotoxic when it was identified as the culprit HDS in at least one qualified hepatotoxicity case.

The HILI cases were adjudicated by the original authors using scoring instruments such as Roussel Uclaf Causality Assessment Method (RUCAM) [56] or diagnosis based on expert opinion [57]. The causality assessment followed the recommendation of the World Health Organization (WHO) and Uppsala Monitoring Centre (UMC) system [73] and was categorized into “certain”, “probable” and “possible”, and “unlikely”. Only the cases with adjudicated causality of “certain”, “probable”, or “possible” were considered.

### 4.3. Other HDS Relevant Data and Standardization

Besides HDS hepatotoxicity, we also collected other relevant data, including but not limited to scientific name, common name, product name, manufacturer, formula, clinical application, toxic ingredients, hepatotoxicity mechanisms, and HDS–drug interactions. In addition, the gender ratio of HDS use and consumption of HDS by ethnicities were also collected. The following websites and databases were used for data collection: Botanical Dietary Supplements from the NIH Office of Dietary Supplements [72], MedlinePlus of the National Library of Medicine [70], Dietary Supplements from the FDA [74], the European Union herbal monograph [71], HerbMed [75], and EMBASE [70].

To facilitate future data analysis and user ease, we attempted to use standard terminologies to categorize and describe HDS. For example, HDS have a broad range of health claims and the EMA has developed 12 categories to describe these HDS applications. Categories for HDS use include circulatory disorders, constipation, cough and cold, eye discomfort, fatigue and weakness, gastrointestinal disorders, loss of appetite, mental stress and mood disorders, mouth and throat disorders, pain and inflammation, skin disorders and minor wounds, and sleep disorders and temporary insomnia. The use categories developed by the EMA were utilized in our database along with two additional categories of HDS use that are common in the United States (i.e., weight loss and bodybuilding).

### 4.4. Development of Web-Based Information Retrieval Tool for HDS

HDS hepatotoxicity data were collected in a Microsoft Excel spreadsheet and then transferred to the Apache Solr system, which is a distributed indexing and load-balanced querying tool. The web-based application was developed using Apache Tomcat as the web server. Hyper Text Markup Language (HTML) code, Cascading Style Sheets (CSS), and Java Server Pages (JSP) were also used in the development of the web application.

The current version of the database supports full-text search of the common name, product name, chemical formula, medical applications, hepatotoxic potential, HDS–drug interaction, and gender preponderance. The HDS hepatotoxicity database is currently used as an internal FDA resource but will soon be open to the public and accessible through the LTKB. The database will continue to be revised and updated annually. To see a screen-shot of the database, refer to Figure 5.

## Supplementary Materials

Supplementary materials can be found at http://www.mdpi.com/1422-0067/19/10/2955/s1.
