# Supplementary material for: The Development of a Database for Herbal and Dietary Supplement Induced Liver Toxicity
Source: Int J Mol Sci. 2018 Sep 28;19(10):2955. doi: 10.3390/ijms19102955 (PMC6213387; doi:10.3390/ijms19102955)
Supplement: Supplementary file 1 [file ijms-19-02955-s001.zip › ijms-344147-suplementary/ijms-344147-Table S1-proofreading.docx]

**Table S1.** The 296 unique HDS and related hepatotoxicity information.

| **Common Name** | **Sources** | **Type** | **Health claims** | **Evidence of HILI** | **Causality Assessment Method and Categories** |
| --- | --- | --- | --- | --- | --- |
| Acai | MedlinePlus | Herb | Weight Control | HILI reported | Expert opinion: probable [1] |
| Aloe Vera | EMA,MedlinePlus | Herb | Gastrointestinal disorders | HILI reported | RUCAM: certain [2,3], probable [2,4,5]  Expert opinion: probable [6–8] |
| Anabolic Steroids | MedlinePlus | hormone | Bodybuilding | HILI reported | RUCAM: certain [9]  Expert opinion: probable [10–14], possible [15] |
| Angelica | Other | Herb | Gastrointestinal disorders | HILI reported | RUCAM: probable [16] |
| Arrowroot | Other | Herb | Gastrointestinal disorders | HILI reported | RUCAM: certain [17] |
| Artemisinin | Other | Herb |  | HILI reported | Expert opinion: probable [18] |
| Atractylis gummifera | Other | Herb | Miscellaneous | HILI reported | Expert opinion: certain [19–21] |
| Bee Pollen | MedlinePlus | Animal | Loss of appetite | HILI reported | Expert opinion: probable [22] |
| Black Cohosh | EMA,MedlinePlus | Herb | Urinary tract and genital disorders | HILI reported | RUCAM: probable [23]  Expert opinion: probable [24–30] |
| Blue-green algae | MedlinePlus | Microbes | Weight Control | HILI reported | Expert opinion: probable [31] |
| Butterbur | MedlinePlus | Herb | Urinary tract and genital disorders | HILI reported | Expert opinion: probable [32,33] |
| Camphor | Other | Herb | Pain and inflammation | HILI reported | Expert opinion: probable [34,35] |
| Cascara | EMA,MedlinePlus | Herb | Constipation | HILI reported | Expert opinion: probable [36,37], possible [38] |
| Centella | EMA | Herb | Mental stress & mood disorders | HILI reported | Expert opinion: certain [39], probable [39,40] |
| Chaparral | Other | Herb | Miscellaneous | HILI reported | NADRPS: probable [41]  Expert opinion: certain [42–47], probable [48,49] |
| Chi R Yun | Other | Herb | Miscellaneous | HILI reported | Expert opinion: certain [50,51] |
| Chromium | MedlinePlus | Vitamin | Bodybuilding | HILI reported | Expert opinion: probable [52,53] |
| Chuan Lian Zi | Other | Herb | Gastrointestinal disorders | HILI reported | RUCAM: probable, possible [54] |
| Clove | EMA,MedlinePlus | Herb | Mouth and throat disorders | HILI reported | Expert opinion: certain [55,56] |
| Colpachi | Other | Herb | Weight Control | HILI reported | NADRPS: possible [57]  Expert opinion: probable [58] |
| Comfrey | EMA | Herb | Pain and inflammation | HILI reported | Expert opinion: certain [59–62], possible [63] |
| Conjugated linoleic acid | MedlinePlus | Vitamin | Weight Control | HILI reported | RUCAM: probable [64], possible [65]  Expert opinion: probable [66] |
| Corydalis | Other | Herb | Mental stress & mood disorders | HILI reported | Expert opinion: certain [67] |
| Creatine | MedlinePlus | Vitamin | Bodybuilding | HILI reported | Expert opinion: possible [68] |
| Echinacea | EMA,MedlinePlus | Herb | Skin disorders & minor wounds, Cough and cold | HILI reported | NADRPS: probable [69]  Expert opinion: probable [70,71] |
| Ephedra | MedlinePlus | Herb | Cough and cold | HILI reported | Expert opinion: certain [72–74], probable [75] |
| European Mistletoe | EMA,MedlinePlus | Herb | Miscellaneous | HILI reported | Expert opinion: certain [76] |
| Flavocoxid | Other | Herb | Pain and inflammation | HILI reported | RUCAM: certain [77], probable [77] |
| Folate | MedlinePlus | Vitamin | Bodybuilding | HILI reported | Expert opinion: probable [78] |
| Garcinia cambogia | MedlinePlus | Herb | Weight Control | HILI reported | RUCAM: probable [79]  Expert opinion: probable [80] |
| Germander | Other | Herb | Weight Control | HILI reported | NADRPS: certain [81], probable [81]  Expert opinion: certain [82–87], probable [84,85,87,88] |
| Glucosamine | MedlinePlus | Vitamin | Miscellaneous | HILI reported | CDS: certain [89], possible [89]  Expert opinion: probable [90], possible [91] |
| Greater Celandine | EMA | Herb | Gastrointestinal disorders | HILI reported | RUCAM, NADRPS: certain [92,93], probable [94]  Expert opinion: probable [95–99] |
| Green Tea | EMA,MedlinePlus | Herb | Fatigue & weakness | HILI reported | RUCAM: certain [100], probable [100,101]  Expert opinion: certain [102–105], probable [106,107], possible [108,109] |
| Gynura segetum | Other | Herb | Pain and inflammation | HILI reported | RUCAM: probable [110,111], possible [110]  Expert opinion: certain [111,112], probable [113] |
| Hovenia dulcis | Other | Herb | Miscellaneous | HILI reported | RUCAM: probable [114] |
| Hydrazine Sulfate | MedlinePlus | Vitamin | Miscellaneous | HILI reported | Expert opinion: probable [115] |
| Impila | Other | Herb | Constipation | HILI reported | Expert opinion: certain [116] |
| Iron | MedlinePlus | Vitamin | Bodybuilding | HILI reported | Expert opinion: certain [117] |
| Jin Bu Huan | Other | Herb | Pain and inflammation | HILI reported | Expert opinion: certain [118–120], probable [121,122], possible [46] |
| Juniper | EMA | Herb | Pain and inflammation,Urinary tract and genital disorders,Gastrointestinal disorders | HILI reported | Expert opinion: certain [123,124] |
| Kava | EMA,MedlinePlus | Herb | Mental stress & mood disorders | HILI reported | CDS, RUCAM, NADRPS: certain [125,126], probable [125,127], possible [125]  Expert opinion: certain [128], probable [129–132], possible [133] |
| Khat | Other | Herb | Mental stress & mood disorders | HILI reported | RUCAM: probable [134]  Expert opinion: probable [135–137] |
| Kombucha tea | Other | Microbes | Miscellaneous | HILI reported | Expert opinion: probable [138,139] |
| Kratom | Other | Herb | Mental stress & mood disorders | HILI reported | Expert opinion: certain [140,141], probable [142] |
| Lesser celandine | Other | Herb | Circulatory disorders | HILI reported | RUCAM: probable [143]  Expert opinion: certain [128] |
| Licorice Root | EMA,MedlinePlus | Herb | Cough and cold | HILI reported | Expert opinion: probable [144] |
| Mao Guo Tian Jie Cai | Other | Herb | Cough and cold | HILI reported | Expert opinion: certain [145] |
| Neem | Other | Herb | Miscellaneous | HILI reported | Expert opinion: certain [146–148] |
| Noni | MedlinePlus | Herb | Miscellaneous | HILI reported | RUCAM: probable [149–154], possible [149]  Expert opinion: probable [155] |
| Pelargonium root | EMA | Herb | Cough and cold | HILI reported | RUCAM: possible [156] |
| Pennyroyal | MedlinePlus | Herb | Cough and cold | HILI reported | Expert opinion: certain [157] |
| Polygonum multiflorum | Other | Herb | Miscellaneous | HILI reported | RUCAM: certain [158–160], probable [159–161] |
| Psoralen | Other | Herb | Skin disorders & minor wounds | HILI reported | RUCAM, NADRPS: probable [162], possible [163]  Expert opinion: probable [164–166] |
| Red Yeast Rice | MedlinePlus | Herb | Circulatory disorders | HILI reported | WHO-UMC, RUCAM: probable [167], possible [167]  Expert opinion: probable [168], possible [169] |
| Reishi Mushroom | MedlinePlus | Microbes | Fatigue & weakness | HILI reported | Expert opinion: probable [170], possible [171] |
| Rhubarb | EMA | Herb | Constipation | HILI reported | RUCAM: possible [54] |
| Saw Palmetto | EMA,MedlinePlus | Herb | Urinary tract and genital disorders | HILI reported | RUCAM: probable [172]  Expert opinion: possible [173] |
| Senna | EMA,MedlinePlus | Herb | Constipation | HILI reported | NADRPS: possible [174]  Expert opinion: certain [175–177], probable [178,179] |
| Siberian Ginseng | EMA,MedlinePlus | Herb | Fatigue & weakness | HILI reported | RUCAM: probable [180], possible [180] |
| Skullcap | Other | Herb | Mental stress & mood disorders | HILI reported | NADRPS, RUCAM: probable [181–183]  Expert opinion: certain [184], probable [26], possible [26,185–187] |
| Sorrel | Other | Herb | Pain and inflammation | HILI reported | Expert opinion: certain [188] |
| St. John's Wort | EMA,MedlinePlus | Herb | Skin disorders & minor wounds,Mental stress & mood disorders,Gastrointestinal disorders | HILI reported | Expert opinion: possible [189,190] |
| Usnic Acid | Other | Herb | Weight Control | HILI reported | Expert opinion: certain [191], probable [73,191–193] |
| Valerian | EMA,MedlinePlus | Herb | Sleep disorders & temporary insomnia,Mental stress & mood disorders | HILI reported | Expert opinion: probable [194–197], possible [26,185,186] |
| Vitamin A | MedlinePlus | Vitamin | Miscellaneous | HILI reported | Expert opinion: certain [198,199] |
| Vitamin B3 | MedlinePlus | Vitamin | Circulatory disorders | HILI reported | WHO-UMC: probable [200]  Expert opinion: probable [201,202] |
| 5-HTP | MedlinePlus | Vitamin | Sleep disorders & temporary insomnia | No HILI reported |  |
| Activated Charcoal | MedlinePlus | Vitamin | Gastrointestinal disorders | No HILI reported |  |
| Agnus Castus Fruit | EMA | Herb | Urinary tract and genital disorders | No HILI reported |  |
| Agrimony | EMA | Herb | Mouth and throat disorders,Skin disorders & minor wounds,Gastrointestinal disorders | No HILI reported |  |
| Alfalfa | MedlinePlus | Herb | Urinary tract and genital disorders | No HILI reported |  |
| American ginseng | MedlinePlus | Herb | Fatigue & weakness | No HILI reported |  |
| Anise | EMA | Herb | Cough and cold,Gastrointestinal disorders | No HILI reported |  |
| Arctic root | EMA | Herb | Fatigue & weakness,Mental stress & mood disorders | No HILI reported |  |
| Aristolochic Acids | MedlinePlus | Herb | Pain and inflammation | No HILI reported |  |
| Arnica | EMA,MedlinePlus | Herb | Pain and inflammation | No HILI reported |  |
| Artichoke | EMA | Herb | Gastrointestinal disorders | No HILI reported |  |
| Ash | EMA | Herb | Pain and inflammation,Urinary tract and genital disorders | No HILI reported |  |
| Ashwagandha | EMA,MedlinePlus | Herb | Mental stress & mood disorders | No HILI reported |  |
| Asian Ginseng | EMA,MedlinePlus | Herb | Fatigue & weakness | No HILI reported |  |
| Astragalus | MedlinePlus | Herb | Gastrointestinal disorders | No HILI reported |  |
| Bacillus coagulans | MedlinePlus | Microbes | Gastrointestinal disorders | No HILI reported |  |
| Bearberry | EMA | Herb | Urinary tract and genital disorders | No HILI reported |  |
| Belladonna | MedlinePlus | Herb | Cough and cold | No HILI reported |  |
| Berberine | MedlinePlus | Herb | Circulatory disorders | No HILI reported |  |
| Bergamot | EMA | Herb | Skin disorders & minor wounds | No HILI reported |  |
| Beta-carotene | MedlinePlus | Herb | Miscellaneous | No HILI reported |  |
| Bifidobacteria | MedlinePlus | Microbes | Gastrointestinal disorders | No HILI reported |  |
| Bilberry | EMA,MedlinePlus | Herb | Mouth and throat disorders,Gastrointestinal disorders,Circulatory disorders | No HILI reported |  |
| Biotin | MedlinePlus | Vitamin | Weight Control | No HILI reported |  |
| Birch | EMA | Herb | Urinary tract and genital disorders | No HILI reported |  |
| Bitter Fennel | EMA | Herb | Urinary tract and genital disorders,Cough and cold,Gastrointestinal disorders | No HILI reported |  |
| Bitter Melon | MedlinePlus | Herb | Gastrointestinal disorders | No HILI reported |  |
| Bitter Orange | MedlinePlus | Herb | Weight Control | No HILI reported |  |
| Bittersweet Nightshade | EMA | Herb | Skin disorders & minor wounds | No HILI reported |  |
| Black psyllium | EMA,MedlinePlus | Herb | Constipation | No HILI reported |  |
| Black tea | MedlinePlus | Herb | Mental stress & mood disorders | No HILI reported |  |
| Blackcurrant | EMA | Herb | Pain and inflammation,Urinary tract and genital disorders | No HILI reported |  |
| Bladderwrack | EMA,MedlinePlus | Herb | Gastrointestinal disorders | No HILI reported |  |
| Blessed thistle | MedlinePlus | Herb | Loss of appetite | No HILI reported |  |
| Blond psyllium | EMA,MedlinePlus | Herb | Constipation | No HILI reported |  |
| Blueberry | MedlinePlus | Herb | Eye discomfort | No HILI reported |  |
| Boldo | EMA | Herb | Gastrointestinal disorders | No HILI reported |  |
| Boron | MedlinePlus | Vitamin | Bodybuilding | No HILI reported |  |
| Bromelain | MedlinePlus | Herb | Pain and inflammation | No HILI reported |  |
| Burdock | EMA | Herb | Loss of appetite,Skin disorders & minor wounds,Urinary tract and genital disorders | No HILI reported |  |
| Butcher's Broom | EMA | Herb | Circulatory disorders | No HILI reported |  |
| Caffeine | MedlinePlus | Vitamin | Weight Control | No HILI reported |  |
| Calcium | MedlinePlus | Vitamin | Bodybuilding | No HILI reported |  |
| Calendula | EMA,MedlinePlus | Herb | Mouth and throat disorders,Skin disorders & minor wounds | No HILI reported |  |
| California poppy | EMA | Herb | Sleep disorders & temporary insomnia,Mental stress & mood disorders | No HILI reported |  |
| Cannabidiol | MedlinePlus | Herb | Mental stress & mood disorders | No HILI reported |  |
| Capsicum | EMA,MedlinePlus | Herb | Pain and inflammation | No HILI reported |  |
| Caraway | EMA | Herb | Gastrointestinal disorders | No HILI reported |  |
| Carnitine | MedlinePlus | Vitamin | Bodybuilding | No HILI reported |  |
| Cartilage (Bovine and Shark) | MedlinePlus | Animal | Fatigue & weakness | No HILI reported |  |
| Castor | EMA | Herb | Gastrointestinal disorders | No HILI reported |  |
| Cat's Claw | EMA,MedlinePlus | Herb | Miscellaneous | No HILI reported |  |
| Centaury | EMA | Herb | Loss of appetite,Gastrointestinal disorders | No HILI reported |  |
| Chamomile | EMA,MedlinePlus | Herb | Mouth and throat disorders,Skin disorders & minor wounds,Cough and cold,Gastrointestinal disorders | No HILI reported |  |
| Chasteberry | MedlinePlus | Herb | Urinary tract and genital disorders | No HILI reported |  |
| Chicory | EMA | Herb | Loss of appetite,Gastrointestinal disorders | No HILI reported |  |
| Chitosan | MedlinePlus | Animal | Weight Control | No HILI reported |  |
| Choline | MedlinePlus | Vitamin | Bodybuilding | No HILI reported |  |
| Chondroitin sulfate | MedlinePlus | Vitamin | Miscellaneous | No HILI reported |  |
| Cinnamon | EMA,MedlinePlus | Herb | Gastrointestinal disorders | No HILI reported |  |
| Coconut | MedlinePlus | Herb | Circulatory disorders | No HILI reported |  |
| Cod Liver Oil | MedlinePlus | Animal | Circulatory disorders | No HILI reported |  |
| Coenzyme Q10 | MedlinePlus | Vitamin | Fatigue & weakness | No HILI reported |  |
| Coleus forskohlii | MedlinePlus | Herb | Weight Control | No HILI reported |  |
| Colloidal Silver | MedlinePlus | Vitamin | Miscellaneous | No HILI reported |  |
| Copper | MedlinePlus | Vitamin | Bodybuilding | No HILI reported |  |
| Couch grass | EMA | Herb | Urinary tract and genital disorders | No HILI reported |  |
| Cranberry | EMA,MedlinePlus | Herb | Urinary tract and genital disorders | No HILI reported |  |
| Crofelemer | Other | Herb | Gastrointestinal disorders | No HILI reported |  |
| Dandelion | EMA,MedlinePlus | Herb | Loss of appetite,Urinary tract and genital disorders,Gastrointestinal disorders | No HILI reported |  |
| Deer Velvet | MedlinePlus | Animal | Circulatory disorders | No HILI reported |  |
| Devil's claw | EMA,MedlinePlus | Herb | Loss of appetite,Pain and inflammation,Gastrointestinal disorders | No HILI reported |  |
| DHEA | MedlinePlus | hormone | Miscellaneous | No HILI reported |  |
| Dittany of Crete herb | EMA | Herb | Skin disorders & minor wounds,Cough and cold,Gastrointestinal disorders | No HILI reported |  |
| Dong quai | EMA,MedlinePlus | Herb | Circulatory disorders | No HILI reported |  |
| Eucalyptus | EMA,MedlinePlus | Herb | Pain and inflammation,Cough and cold | No HILI reported |  |
| European Elder | EMA,MedlinePlus | Herb | Cough and cold | No HILI reported |  |
| European Goldenrod | EMA | Herb | Urinary tract and genital disorders | No HILI reported |  |
| Evening Primrose | EMA,MedlinePlus | Herb | Skin disorders & minor wounds | No HILI reported |  |
| Eyebright | EMA | Herb | Mouth and throat disorders | No HILI reported |  |
| Fenugreek | EMA,MedlinePlus | Herb | Loss of appetite,Skin disorders & minor wounds | No HILI reported |  |
| Feverfew | EMA,MedlinePlus | Herb | Pain and inflammation | No HILI reported |  |
| Flaxseed | EMA,MedlinePlus | Herb | Constipation | No HILI reported |  |
| Fluoride | MedlinePlus | Vitamin | Bodybuilding | No HILI reported |  |
| Frangula | EMA | Herb | Constipation | No HILI reported |  |
| Fucoxanthin | MedlinePlus | Vitamin | Weight Control | No HILI reported |  |
| Fumitory | EMA | Herb | Gastrointestinal disorders | No HILI reported |  |
| Garlic | EMA,MedlinePlus | Herb | Circulatory disorders | No HILI reported |  |
| Gelatin | MedlinePlus | Animal | Weight Control | No HILI reported |  |
| Gentian | EMA | Herb | Gastrointestinal disorders | No HILI reported |  |
| Ginger | EMA,MedlinePlus | Herb | Gastrointestinal disorders | No HILI reported |  |
| Ginkgo | EMA,MedlinePlus | Herb | Circulatory disorders | No HILI reported |  |
| Glucomannan | MedlinePlus | Herb | Weight Control | No HILI reported |  |
| Goji | MedlinePlus | Herb | Circulatory disorders | No HILI reported |  |
| Goldenseal | MedlinePlus | Herb | Cough and cold | No HILI reported |  |
| Grape | EMA,MedlinePlus | Herb | Circulatory disorders | No HILI reported |  |
| Green Coffee | MedlinePlus | Herb | Weight Control | No HILI reported |  |
| Guar gum | MedlinePlus | Herb | Weight Control | No HILI reported |  |
| Guarana | EMA,MedlinePlus | Herb | Fatigue & weakness | No HILI reported |  |
| Gumweed herb | EMA | Herb | Cough and cold | No HILI reported |  |
| Gymnema | MedlinePlus | Herb | Miscellaneous | No HILI reported |  |
| Hamamelis | EMA | Herb | Mouth and throat disorders,Skin disorders & minor wounds,Circulatory disorders, Eye discomfort | No HILI reported |  |
| Hawthorn | EMA,MedlinePlus | Herb | Circulatory disorders,Mental stress & mood disorders | No HILI reported |  |
| Hedge mustard | EMA | Herb | Mouth and throat disorders | No HILI reported |  |
| Hibiscus | MedlinePlus | Herb | Loss of appetite | No HILI reported |  |
| Honey | MedlinePlus | Animal | Cough and cold | No HILI reported |  |
| Hoodia | MedlinePlus | Herb | Weight Control | No HILI reported |  |
| Hops | EMA,MedlinePlus | Herb | Loss of appetite,Sleep disorders & temporary insomnia,Mental stress & mood disorders | No HILI reported |  |
| Horny Goat Weed | MedlinePlus | Herb | Miscellaneous | No HILI reported |  |
| Horse Chestnut | EMA,MedlinePlus | Herb | Urinary tract and genital disorders,Circulatory disorders | No HILI reported |  |
| Horsetail | EMA,MedlinePlus | Herb | Urinary tract and genital disorders | No HILI reported |  |
| Hyssop | Other | Herb | Gastrointestinal disorders | No HILI reported |  |
| Iceland moss | EMA | Herb | Loss of appetite,Mouth and throat disorders,Cough and cold | No HILI reported |  |
| Iodine | MedlinePlus | Vitamin | Urinary tract and genital disorders | No HILI reported |  |
| Ironwort | EMA | Herb | Cough and cold,Gastrointestinal disorders | No HILI reported |  |
| Ivy | EMA | Herb | Cough and cold | No HILI reported |  |
| Java Tea | EMA | Herb | Urinary tract and genital disorders | No HILI reported |  |
| Javanese turmeric | EMA | Herb | Gastrointestinal disorders | No HILI reported |  |
| Kalmegh | EMA | Herb | Cough and cold | No HILI reported |  |
| Katula | EMA | Herb | Gastrointestinal disorders | No HILI reported |  |
| Knotgrass herb | EMA | Herb | Mouth and throat disorders,Urinary tract and genital disorders,Cough and cold | No HILI reported |  |
| Kola nut | EMA,MedlinePlus | Herb | Fatigue & weakness | No HILI reported |  |
| Lactobacillus | MedlinePlus | Microbes | Gastrointestinal disorders | No HILI reported |  |
| L-arginine | MedlinePlus | Vitamin | Gastrointestinal disorders | No HILI reported |  |
| Lavender | EMA,MedlinePlus | Herb | Sleep disorders & temporary insomnia,Mental stress & mood disorders | No HILI reported |  |
| Lime flower | EMA | Herb | Cough and cold,Mental stress & mood disorders | No HILI reported |  |
| Lipase | MedlinePlus | Herb | Gastrointestinal disorders | No HILI reported |  |
| Lovage | EMA | Herb | Urinary tract and genital disorders | No HILI reported |  |
| L-tryptophan | MedlinePlus | Vitamin | Sleep disorders & temporary insomnia | No HILI reported |  |
| Lutein | MedlinePlus | Vitamin | Eye discomfort | No HILI reported |  |
| Lycopene | MedlinePlus | Vitamin | Circulatory disorders | No HILI reported |  |
| Maca | MedlinePlus | Herb | Circulatory disorders | No HILI reported |  |
| Magnesium | MedlinePlus | Vitamin | Bodybuilding | No HILI reported |  |
| Majoram | EMA | Herb | Skin disorders & minor wounds,Gastrointestinal disorders | No HILI reported |  |
| Malabar-nut | EMA | Herb | Mouth and throat disorders | No HILI reported |  |
| Mallow | EMA | Herb | Mouth and throat disorders | No HILI reported |  |
| Manganese | MedlinePlus | Vitamin | Bodybuilding | No HILI reported |  |
| Mangosteen | MedlinePlus | Herb | Urinary tract and genital disorders | No HILI reported |  |
| Marshmallow | EMA | Herb | Mouth and throat disorders,Cough and cold,Gastrointestinal disorders | No HILI reported |  |
| Mastic tree resin | EMA | Herb | Gastrointestinal disorders | No HILI reported |  |
| Meadowsweet | EMA | Herb | Pain and inflammation,Cough and cold | No HILI reported |  |
| Melatonin | MedlinePlus | hormone | Sleep disorders & temporary insomnia | No HILI reported |  |
| Melilot | EMA | Herb | Pain and inflammation,Skin disorders & minor wounds,Circulatory disorders | No HILI reported |  |
| Melissa | EMA | Herb | Sleep disorders & temporary insomnia,Mental stress & mood disorders,Gastrointestinal disorders | No HILI reported |  |
| Methylsulfonylmethane | MedlinePlus | Vitamin | Pain and inflammation | No HILI reported |  |
| Milk Thistle | EMA,MedlinePlus | Herb | Gastrointestinal disorders | No HILI reported |  |
| Motherwort | EMA | Herb | Circulatory disorders,Mental stress & mood disorders | No HILI reported |  |
| Mouse-ear hawkweed | EMA | Herb | Urinary tract and genital disorders | No HILI reported |  |
| Mullein | EMA | Herb | Mouth and throat disorders,Cough and cold | No HILI reported |  |
| Myrrh | EMA | Herb | Mouth and throat disorders,Skin disorders & minor wounds | No HILI reported |  |
| Nettle | EMA | Herb | Pain and inflammation,Skin disorders & minor wounds,Urinary tract and genital disorders | No HILI reported |  |
| Oak Bark | EMA | Herb | Skin disorders & minor wounds,Circulatory disorders,Gastrointestinal disorders | No HILI reported |  |
| Oats | EMA,MedlinePlus | Herb | Skin disorders & minor wounds,Sleep disorders & temporary insomnia,Mental stress & mood disorders | No HILI reported |  |
| Olive | EMA,MedlinePlus | Herb | Urinary tract and genital disorders | No HILI reported |  |
| Omega-3 Fatty Acids | MedlinePlus | Animal | Circulatory disorders | No HILI reported |  |
| Omega-6 Fatty Acids | MedlinePlus | Vitamin | Circulatory disorders | No HILI reported |  |
| Onion | EMA | Herb | Loss of appetite | No HILI reported |  |
| Oregano | MedlinePlus | Herb | Cough and cold | No HILI reported |  |
| Palm Oil | MedlinePlus | Herb | Weight Control | No HILI reported |  |
| Papaya | MedlinePlus | Herb | Gastrointestinal disorders | No HILI reported |  |
| Passion Flower | EMA | Herb | Sleep disorders & temporary insomnia,Mental stress & mood disorders | No HILI reported |  |
| Pau D'arco | MedlinePlus | Herb | Cough and cold | No HILI reported |  |
| Peanut Oil | MedlinePlus | Herb | Circulatory disorders | No HILI reported |  |
| Peony | EMA | Herb | Pain and inflammation | No HILI reported |  |
| Peppermint | EMA,MedlinePlus | Herb | Pain and inflammation,Skin disorders & minor wounds,Cough and cold,Gastrointestinal disorders | No HILI reported |  |
| Peru balsam | EMA | Herb | Urinary tract and genital disorders | No HILI reported |  |
| Phosphate salts | MedlinePlus | Vitamin | Circulatory disorders | No HILI reported |  |
| Phosphorus | MedlinePlus | Vitamin | Bodybuilding | No HILI reported |  |
| Pink rock-rose | EMA | Herb | Mental stress & mood disorders | No HILI reported |  |
| Podophyllum | Other | Herb | Constipation | No HILI reported |  |
| Polypody | EMA | Herb | Cough and cold,Constipation | No HILI reported |  |
| Pomegranate | MedlinePlus | Herb | Miscellaneous | No HILI reported |  |
| Potassium | MedlinePlus | Vitamin | Bodybuilding | No HILI reported |  |
| Primula | EMA | Herb | Cough and cold | No HILI reported |  |
| Probiotics | MedlinePlus | Microbes | Gastrointestinal disorders | No HILI reported |  |
| Propolis | MedlinePlus | Animal | Mouth and throat disorders | No HILI reported |  |
| Pumpkin seed | EMA | Herb | Urinary tract and genital disorders | No HILI reported |  |
| Pygeum africanum | EMA | Herb | Urinary tract and genital disorders | No HILI reported |  |
| Pyruvate | MedlinePlus | Vitamin | Weight Control | No HILI reported |  |
| Raspberry | EMA | Herb | Mouth and throat disorders,Pain and inflammation,Gastrointestinal disorders | No HILI reported |  |
| Raspberry Ketone | MedlinePlus | Herb | Weight Control | No HILI reported |  |
| Red Clover | MedlinePlus | Herb | Urinary tract and genital disorders | No HILI reported |  |
| Restharrow | EMA | Herb | Urinary tract and genital disorders | No HILI reported |  |
| Resveratrol | MedlinePlus | Vitamin | Circulatory disorders | No HILI reported |  |
| Ribwort Plantain | EMA | Herb | Cough and cold | No HILI reported |  |
| Roman chamomile | EMA,MedlinePlus | Herb | Gastrointestinal disorders | No HILI reported |  |
| Rose Hip | EMA,MedlinePlus | Herb | Mouth and throat disorders,Skin disorders & minor wounds | No HILI reported |  |
| Rosemary | EMA | Herb | Pain and inflammation,Circulatory disorders,Gastrointestinal disorders | No HILI reported |  |
| Saccharomyces boulardii | EMA,MedlinePlus | Microbes | Miscellaneous | No HILI reported |  |
| Sage | EMA,MedlinePlus | Herb | Mouth and throat disorders,Skin disorders & minor wounds,Gastrointestinal disorders | No HILI reported |  |
| S-adenosylmethionine | MedlinePlus | Vitamin | Miscellaneous | No HILI reported |  |
| Sandy everlasting | EMA | Herb | Gastrointestinal disorders | No HILI reported |  |
| Sassafras | Other | Herb | Urinary tract and genital disorders | No HILI reported |  |
| Selenium | MedlinePlus | Vitamin | Bodybuilding | No HILI reported |  |
| Shepherds Purse | EMA | Herb | Urinary tract and genital disorders | No HILI reported |  |
| Silver lime flower | EMA | Herb | Cough and cold | No HILI reported |  |
| Slippery Elm | MedlinePlus | Herb | Circulatory disorders | No HILI reported |  |
| Soy | EMA,MedlinePlus | Herb | Skin disorders & minor wounds, Fatigue & weakness | No HILI reported |  |
| Spearmint | MedlinePlus | Herb | Gastrointestinal disorders | No HILI reported |  |
| Stevia | MedlinePlus | Herb | Gastrointestinal disorders | No HILI reported |  |
| Tea Tree Oil | EMA,MedlinePlus | Herb | Mouth and throat disorders,Skin disorders & minor wounds | No HILI reported |  |
| Three-lobed sage leaf | EMA | Herb | Mouth and throat disorders | No HILI reported |  |
| Thunder God Vine | MedlinePlus | Herb | Pain and inflammation | No HILI reported |  |
| Thyme | EMA | Herb | Urinary tract and genital disorders, Cough and cold | No HILI reported |  |
| Tormentil | EMA | Herb | Mouth and throat disorders,Gastrointestinal disorders | No HILI reported |  |
| Turmeric | EMA,MedlinePlus | Herb | Gastrointestinal disorders | No HILI reported |  |
| Vitamin B1 | MedlinePlus | Vitamin | Miscellaneous | No HILI reported |  |
| Vitamin B12 | MedlinePlus | Vitamin | Bodybuilding | No HILI reported |  |
| Vitamin B2 | MedlinePlus | Vitamin | Miscellaneous | No HILI reported |  |
| Vitamin B5 | MedlinePlus | Vitamin | Miscellaneous | No HILI reported |  |
| Vitamin C | MedlinePlus | Vitamin | Miscellaneous | No HILI reported |  |
| Vitamin D | MedlinePlus | Vitamin | Miscellaneous | No HILI reported |  |
| Vitamin E | MedlinePlus | Vitamin | Miscellaneous | No HILI reported |  |
| Vitamin K | MedlinePlus | Vitamin | Circulatory disorders | No HILI reported |  |
| Vitamin B6 | MedlinePlus | Vitamin | Urinary tract and genital disorders | No HILI reported |  |
| Walnut | EMA | Herb | Skin disorders & minor wounds | No HILI reported |  |
| Whey Protein | MedlinePlus | Animal | Weight Control | No HILI reported |  |
| White horehound | EMA | Herb | Loss of appetite,Cough and cold,Gastrointestinal disorders | No HILI reported |  |
| White kidney bean | EMA,MedlinePlus | Herb | Urinary tract and genital disorders | No HILI reported |  |
| Wild Pansy | EMA | Herb | Skin disorders & minor wounds | No HILI reported |  |
| Wild Strawberry | EMA | Herb | Gastrointestinal disorders | No HILI reported |  |
| Wild yam | MedlinePlus | Herb | Urinary tract and genital disorders | No HILI reported |  |
| Willow Bark | EMA,MedlinePlus | Herb | Pain and inflammation,Cough and cold | No HILI reported |  |
| Willow herb | EMA | Herb | Urinary tract and genital disorders | No HILI reported |  |
| Wormwood | EMA | Herb | Loss of appetite,Gastrointestinal disorders | No HILI reported |  |
| Yarrow | EMA | Herb | Loss of appetite,Skin disorders & minor wounds,Urinary tract and genital disorders,Gastrointestinal disorders | No HILI reported |  |
| Yerba Mate | EMA,MedlinePlus | Herb | Fatigue & weakness,Urinary tract and genital disorders | No HILI reported |  |
| Yohimbe | MedlinePlus | Herb | Miscellaneous | No HILI reported |  |
| Zinc | MedlinePlus | Vitamin | Miscellaneous | No HILI reported |  |

*Abbreviation: HILI, herbal and dietary supplements induced liver injury; ALF, acute liver failure. EMA, European Medicine Agency; RUCAM, Roussel Uclaf Causality Assessment Method. NADRPS, Naranjo Adverse Reactions Probability Scale. CDS, Clinical Diagnostic Scale. WHO-UMC, the causality assessment system proposed by World Health Organization Collaborating Centre for International Drug Monitoring, the Uppsala Monitoring Center.

**Reference**

1. Stratton, L.; McNeilly, J.; #039; Neill, C.; Cash, W.J. Acai berry induced cholestatic jaundice. *J. Med. Cases* **2014**, *5*, 373–375.
2. Yang, H.N.; Kim, D.J.; Kim, Y.M.; Kim, B.H.; Sohn, K.M.; Choi, M.J.; Choi, Y.H. Aloe-induced toxic hepatitis. *J. Korean Med. Sci.* **2010**, *25*, 492–495.
3. Lee, J.; Lee, M.S.; Nam, K.W. Acute toxic hepatitis caused by an aloe vera preparation in a young patient: A case report with a literature review. *Korean J. Gastroenterol.* **2014**, *64*, 54–58.
4. Bottenberg, M.M.; Wall, G.C.; Harvey, R.L.; Habib, S. Oral aloe vera-induced hepatitis. *Ann. Pharmacother.* **2007**, *41*, 1740–1743.
5. Curciarello, J.; De Ortuzar, S.; Borzi, S.; Bosia, D. Severe acute hepatitis associated with intake of aloe vera tea. *Gastroenterol Hepatol* **2008**, *31*, 436–438.
6. Rabe, C.; Musch, A.; Schirmacher, P.; Kruis, W.; Hoffmann, R. Acute hepatitis induced by an aloe vera preparation: A case report. *World J. Gastroenterol.* **2005**, *11*, 303–304.
7. Kanat, O.; Ozet, A.; Ataergin, S. Aloe vera-induced acute toxic hepatitis in a healthy young man. *Eur. J. Intern. Med.* **2006**, *17*, 589.
8. Parlati, L.; Voican, C.S.; Perlemuter, K.; Perlemuter, G. Aloe vera-induced acute liver injury: A case report and literature review. *Clin. Res. Hepatol. Gastroenterol.* **2017**, *41*, e39-e42.
9. Vilella, A.L.; Limsuwat, C.; Williams, D.R.; Seifert, C.F. Cholestatic jaundice as a result of combination designer supplement ingestion. *Ann. Pharmacother.* **2013**, *47*, e33.
10. Rosenfeld, G.A.; Chang, A.; Poulin, M.; Kwan, P.; Yoshida, E. Cholestatic jaundice, acute kidney injury and acute pancreatitis secondary to the recreational use of methandrostenolone: A case report. *J. Med. Case Rep.* **2011**, *5*, 138.
11. Krishnan, P.V.; Feng, Z.Z.; Gordon, S.C. Prolonged intrahepatic cholestasis and renal failure secondary to anabolic androgenic steroid-enriched dietary supplements. *J. Clin. Gastroenterol.* **2009**, *43*, 672–675.
12. Elsharkawy, A.M.; McPherson, S.; Masson, S.; Burt, A.D.; Dawson, R.T.; Hudson, M. Cholestasis secondary to anabolic steroid use in young men. *BMJ* **2012**, *344*, e468.
13. Hymel, B.M.; Victor, D.W.; Alvarez, L.; Shores, N.J.; Balart, L.A. Mastabol induced acute cholestasis: A case report. *World J. Hepatol* **2013**, *5*, 133–136.
14. El Sherrif, Y.; Potts, J.R.; Howard, M.R.; Barnardo, A.; Cairns, S.; Knisely, A.S.; Verma, S. Hepatotoxicity from anabolic androgenic steroids marketed as dietary supplements: Contribution from atp8b1/abcb11 mutations? *Liver Int.* **2013**, *33*, 1266–1270.
15. Wingert, N.; Tavakoli, H.; Yoder, E. Acute hepatitis and personality change in a 31-year-old man taking prohormone supplement sus500. *Psychosomatics* **2010**, *51*, 340–344.
16. Bjornsson, E.S.; Bergmann, O.M.; Bjornsson, H.K.; Kvaran, R.B.; Olafsson, S. Incidence, presentation, and outcomes in patients with drug-induced liver injury in the general population of iceland. *Gastroenterology* **2013**, *144*, 1419–1425.
17. Kim, S.Y.; Yim, H.J.; Ahn, J.H.; Kim, J.H.; Kim, J.N.; Yoon, I.; Kim, D.I.; Lee, H.S.; Lee, S.W.; Choi, J.H. Two cases of toxic hepatitis caused by arrowroot juice. *Korean J. Hepatol.* **2009**, *15*, 504–509.
18. Centers for Disease, C.; Prevention. Hepatitis temporally associated with an herbal supplement containing artemisinin-washington, 2008. *Morb. Mortal. Wkly. Rep.* **2009**, *58*, 854–856.
19. Georgiou, M.; Sianidou, L.; Hatzis, T.; Papadatos, J.; Koutselinis, A. Hepatotoxicity due to atractylis gummifera-l. *J. Toxicol. Clin. Toxicol.* **1988**, *26*, 487–493.
20. Bouziri, A.; Hamdi, A.; Menif, K.; Ben Jaballah, N. Hepatorenal injury induced by cutaneous application of atractylis gummifera l. *Clin. Toxicol. (Phila)* **2010**, *48*, 752–754.
21. Mouaffak, Y.; Boutbaoucht, M.; Ejlaidi, A.; Toufiki, R.; Younous, S. fatal poisoning by atractylis gummifera l.: A case report. *Arch Pediatr* **2013**, *20*, 496–498.
22. Shad, J.A.; Chinn, C.G.; Brann, O.S. Acute hepatitis after ingestion of herbs. *South Med. J.* **1999**, *92*, 1095–1097.
23. Lim, T.Y.; Considine, A.; Quaglia, A.; Shawcross, D.L. Subacute liver failure secondary to black cohosh leading to liver transplantation. *BMJ Case Rep.* **2013**, *2013*.
24. Levitsky, J.; Alli, T.A.; Wisecarver, J.; Sorrell, M.F. Fulminant liver failure associated with the use of black cohosh. *Dig. Dis. Sci.* **2005**, *50*, 538–539.
25. Chow, E.C.; Teo, M.; Ring, J.A.; Chen, J.W. Liver failure associated with the use of black cohosh for menopausal symptoms. *Med. J. Aust.* **2008**, *188*, 420–422.
26. Whiting, P.W.; Clouston, A.; Kerlin, P. Black cohosh and other herbal remedies associated with acute hepatitis. *Med. J. Aust.* **2002**, *177*, 440–443.
27. Cohen, S.M.; O'Connor, A.M.; Hart, J.; Merel, N.H.; Te, H.S. Autoimmune hepatitis associated with the use of black cohosh: A case study. *Menopause* **2004**, *11*, 575–577.
28. Joy, D.; Joy, J.; Duane, P. Black cohosh: A cause of abnormal postmenopausal liver function tests. *Climacteric* **2008**, *11*, 84–88.
29. Nisbet, B.C.; O'Connor, R.E. Black cohosh-induced hepatitis. *Del. Med. J.* **2007**, *79*, 441–444.
30. Muqeet Adnan, M.; Khan, M.; Hashmi, S.; Hamza, M.; AbdulMujeeb, S.; Amer, S. Black cohosh and liver toxicity: Is there a relationship? *Case Rep. Gastrointest. Med.* **2014**, *2014*, 860614.
31. Iwasa, M.; Yamamoto, M.; Tanaka, Y.; Kaito, M.; Adachi, Y. Spirulina-associated hepatotoxicity. *Am. J. Gastroenterol.* **2002**, *97*, 3212–3213.
32. Kalin, P. The common butterbur (petasites hybridus)—portrait of a medicinal herb. *Forsch Komplementarmed Klass Naturheilkd* **2003**, *10*, 41–44.
33. Anderson, N.; Meier, T.; Borlak, J. Toxicogenomics applied to cultures of human hepatocytes enabled an identification of novel petasites hybridus extracts for the treatment of migraine with improved hepatobiliary safety. *Toxicol. Sci.* **2009**, *112*, 507–520.
34. Uc, A.; Bishop, W.P.; Sanders, K.D. Camphor hepatotoxicity. *South Med. J.* **2000**, *93*, 596–598.
35. Rampini, S.K.; Schneemann, M.; Rentsch, K.; Bachli, E.B. Camphor intoxication after cao gio (coin rubbing). *JAMA* **2002**, *288*, 45.
36. Jacobsen, C.; Semb, S.; Kromann-Andersen, H. toxic hepatitis following consumption of the herbal medicinal product cascara sagrada. *Ugeskr Laeger* **2009**, *171*, 3367–3369.
37. Nakasone, E.S.; Tokeshi, J. A serendipitous find: A case of cholangiocarcinoma identified incidentally after acute liver injury due to cascara sagrada ingestion. *Hawaii J. Med. Public Health* **2015**, *74*, 200–202.
38. Nadir, A.; Reddy, D.; van Thiel, D.H. Cascara sagrada-induced intrahepatic cholestasis causing portal hypertension: Case report and review of herbal hepatotoxicity. *Am. J. Gastroenterol.* **2000**, *95*, 3634–3637.
39. Jorge, O.A.; Jorge, A.D. Hepatotoxicity associated with the ingestion of centella asiatica. *Rev. Esp. Enferm. Dig.* **2005**, *97*, 115–124.
40. Dantuluri, S.; North-Lewis, P.; Karthik, S.V. Gotu kola induced hepatotoxicity in a child—need for caution with alternative remedies. *Dig. Liver Dis.* **2011**, *43*, 500.
41. Grant, K.L.; Boyer, L.V.; Erdman, B.E. Chaparral-induced hepatotoxicity. *Integr. Med.* **1998**, *1*, 83–87.
42. Katz, M.; Saibil, F. Herbal hepatitis: Subacute hepatic necrosis secondary to chaparral leaf. *J. Clin. Gastroenterol.* **1990**, *12*, 203–206.
43. Batchelor, W.B.; Heathcote, J.; Wanless, I.R. Chaparral-induced hepatic injury. *Am. J. Gastroenterol.* **1995**, *90*, 831–833.
44. Alderman, S.; Kailas, S.; Goldfarb, S.; Singaram, C.; Malone, D.G. Cholestatic hepatitis after ingestion of chaparral leaf: Confirmation by endoscopic retrograde cholangiopancreatography and liver biopsy. *J. Clin. Gastroenterol.* **1994**, *19*, 242–247.
45. Gordon, D.W.; Rosenthal, G.; Hart, J.; Sirota, R.; Baker, A.L. Chaparral ingestion. The broadening spectrum of liver injury caused by herbal medications. *JAMA* **1995**, *273*, 489–490.
46. Haller, C.A.; Dyer, J.E.; Ko, R.; Olson, K.R. Evidence-Based Case Reviews: Making a diagnosis of herbal-related toxic hepatitis. *West. J. Med.* **2002**, *176*, 39–44.
47. Kauma, H.; Koskela, R.; Makisalo, H.; Autio-Harmainen, H.; Lehtola, J.; Hockerstedt, K. Toxic acute hepatitis and hepatic fibrosis after consumption of chaparral tablets. *Scand. J. Gastroenterol.* **2004**, *39*, 1168–1171.
48. Centers for Disease, C. Chaparral-induced toxic hepatitis--california and texas, 1992. *Morb. Mortal. Wkly. Rep.* **1992**, *41*, 812–814.
49. Smith, B.C.; Desmond, P.V. Acute hepatitis induced by ingestion of the herbal medication chaparral. *Aust. N. Z. J. Med.* **1993**, *23*, 526.
50. Lin, T.J.; Tsai, M.S.; Chiou, N.M.; Deng, J.F.; Chiu, N.Y. Hepatotoxicity caused by breynia officinalis. *Vet. Hum. Toxicol.* **2002**, *44*, 87–88.
51. Lin, T.J.; Su, C.C.; Lan, C.K.; Jiang, D.D.; Tsai, J.L.; Tsai, M.S. Acute poisonings with breynia officinalis--an outbreak of hepatotoxicity. *J. Toxicol. Clin. Toxicol.* **2003**, *41*, 591–594.
52. Cerulli, J.; Grabe, D.W.; Gauthier, I.; Malone, M.; McGoldrick, M.D. Chromium picolinate toxicity. *Ann. Pharmacother.* **1998**, *32*, 428–431.
53. Lanca, S.; Alves, A.; Vieira, A.I.; Barata, J.; de Freitas, J.; de Carvalho, A. Chromium-induced toxic hepatitis. *Eur. J. Intern. Med.* **2002**, *13*, 518–520.
54. Melchart, D.; Hager, S.; Albrecht, S.; Dai, J.; Weidenhammer, W.; Teschke, R. Herbal traditional chinese medicine and suspected liver injury: A prospective study. *World J. Hepatol.* **2017**, *9*, 1141–1157.
55. Hartnoll, G.; Moore, D.; Douek, D. Near fatal ingestion of oil of cloves. *Arch. Dis. Child* **1993**, *69*, 392–393.
56. Eisen, J.S.; Koren, G.; Juurlink, D.N.; Ng, V.L. N-acetylcysteine for the treatment of clove oil-induced fulminant hepatic failure. *J. Toxicol. Clin. Toxicol.* **2004**, *42*, 89–92.
57. Wurtz, A.S.; Vial, T.; Isoard, B.; Saillard, E. Possible hepatotoxicity from copaltra, an herbal medicine. *Ann. Pharmacother.* **2002**, *36*, 941–942.
58. Bruguera, M.; Herrera, S.; Lazaro, E.; Madurga, M.; Navarro, M.; de Abajo, F.J. acute hepatitis associated with colpachi intake. Apropros of 5 cases. *Gastroenterol. Hepatol.* **2007**, *30*, 66–68.
59. Yeong, M.L.; Swinburn, B.; Kennedy, M.; Nicholson, G. Hepatic veno-occlusive disease associated with comfrey ingestion. *J. Gastroenterol. Hepatol.* **1990**, *5*, 211–214.
60. Bach, N.; Thung, S.N.; Schaffner, F. Comfrey herb tea-induced hepatic veno-occlusive disease. *Am. J. Med.* **1989**, *87*, 97–99.
61. Weston, C.F.; Cooper, B.T.; Davies, J.D.; Levine, D.F. Veno-occlusive disease of the liver secondary to ingestion of comfrey. *Br. Med. J. (Clin. Res. Ed.)* **1987**, *295*, 183.
62. Ridker, P.M.; Ohkuma, S.; McDermott, W.V.; Trey, C.; Huxtable, R.J. Hepatic venocclusive disease associated with the consumption of pyrrolizidine-containing dietary supplements. *Gastroenterology* **1985**, *88*, 1050–1054.
63. Miskelly, F.G.; Goodyer, L.I. Hepatic and pulmonary complications of herbal medicines. *Postgrad. Med. J.* **1992**, *68*, 935.
64. Ramos, R.; Mascarenhas, J.; Duarte, P.; Vicente, C.; Casteleiro, C. Conjugated linoleic acid-induced toxic hepatitis: First case report. *Dig. Dis. Sci.* **2009**, *54*, 1141–1143.
65. Nortadas, R.; Barata, J. Fulminant hepatitis during self-medication with conjugated linoleic acid. *Ann. Hepatol.* **2012**, *11*, 265–267.
66. Bilal, M.; Patel, Y.; Burkitt, M.; Babich, M. Linoleic acid induced acute hepatitis: A case report and review of the literature. *Case Rep. Hepatol.* **2015**, *2015*, 807354.
67. Kang, H.S.; Choi, H.S.; Yun, T.J.; Lee, K.G.; Seo, Y.S.; Yeon, J.E.; Byun, K.S.; Um, S.H.; Kim, C.D.; Ryu, H.S. a case of acute cholestatic hepatitis induced by corydalis speciosa max. *Korean J. Hepatol.* **2009**, *15*, 517–523.
68. Whitt, K.N.; Ward, S.C.; Deniz, K.; Liu, L.; Odin, J.A.; Qin, L. Cholestatic liver injury associated with whey protein and creatine supplements. *Semin. Liver Dis.* **2008**, *28*, 226–231..
69. Gabranis, I.; Koufakis, T.; Papakrivos, I.; Batala, S. Echinacea-associated acute cholestatic hepatitis. *J. Postgrad. Med.* **2015**, *61*, 211–212.
70. Lawrenson, J.A.; Walls, T.; Day, A.S. Echinacea-induced acute liver failure in a child. *J. Paediatr. Child Health* **2014**, *50*, 841.
71. Kocaman, O.; Hulagu, S.; Senturk, O. Echinacea-induced severe acute hepatitis with features of cholestatic autoimmune hepatitis. *Eur. J. Intern. Med.* **2008**, *19*, 148.
72. Nadir, A.; Agrawal, S.; King, P.D.; Marshall, J.B. Acute hepatitis associated with the use of a chinese herbal product, ma-huang. *Am. J. Gastroenterol.* **1996**, *91*, 1436–1438.
73. Neff, G.W.; Reddy, K.R.; Durazo, F.A.; Meyer, D.; Marrero, R.; Kaplowitz, N. Severe hepatotoxicity associated with the use of weight loss diet supplements containing ma huang or usnic acid. *J. Hepatol.* **2004**, *41*, 1062–1064.
74. Skoulidis, F.; Alexander, G.J.; Davies, S.E. Ma huang associated acute liver failure requiring liver transplantation. *Eur. J. Gastroenterol. Hepatol.* **2005**, *17*, 581–584.
75. Charalampopoulos, A.; Karatsourakis, T.; Tsiodra, P. Acute hepatitis associated with the use of ma-huang in a young adult. *Eur J Intern Med* **2007**, *18*, 81.
76. Harvey, J.; Colin-Jones, D.G. Mistletoe hepatitis. *Br. Med. J. (Clin. Res. Ed.)* **1981**, *282*, 186–187.
77. Chalasani, N.; Vuppalanchi, R.; Navarro, V.; Fontana, R.; Bonkovsky, H.; Barnhart, H.; Kleiner, D.E.; Hoofnagle, J.H. Acute liver injury due to flavocoxid (limbrel), a medical food for osteoarthritis: A case series. *Ann. Intern. Med.* **2012**, *156*, 857–860, W297–W300.
78. Matsubara, S.; Imai, K.; Murayama, K.; Higashizawa, T. Severe liver dysfunction during nausea and vomiting of pregnancy: Folic acid supplement as a suggested culprit. *J. Obstet. Gynaecol.* **2012**, *32*, 701–702.
79. Corey, R.; Werner, K.T.; Singer, A.; Moss, A.; Smith, M.; Noelting, J.; Rakela, J. Acute liver failure associated with garcinia cambogia use. *Ann. Hepatol.* **2016**, *15*, 123–126.
80. Melendez-Rosado, J.; Snipelisky, D.; Matcha, G.; Stancampiano, F. Acute hepatitis induced by pure garcinia cambogia. *J. Clin. Gastroenterol.* **2015**, *49*, 449–450.
81. Gori, L.; Galluzzi, P.; Mascherini, V.; Gallo, E.; Lapi, F.; Menniti-Ippolito, F.; Raschetti, R.; Mugelli, A.; Vannacci, A.; Firenzuoli, F. Two contemporary cases of hepatitis associated with teucrium chamaedrys l. Decoction use: Case reports and review of literature. *Basic Clin. Pharmacol. Toxicol.* **2011**, *109*, 521–526.
82. Mostefa-Kara, N.; Pauwels, A.; Pines, E.; Biour, M.; Levy, V.G. Fatal hepatitis after herbal tea. *Lancet* **1992**, *340*, 674.
83. Legoux, J.L.; Maitre, F.; Labarriere, D.; Gargot, D.; Festin, D.; Causse, X. Cytolytic hepatitis and wild germander: A new case with reintroduction. *Gastroenterol. Clin. Biol.* **1992**, *16*, 813–815.
84. Pauwels, A.; Thierman-Duffaud, D.; Azanowsky, J.M.; Loiseau, D.; Biour, M.; Levy, V.G. acute hepatitis caused by wild germander. Hepatotoxicity of herbal remedies. Two cases. *Gastroenterol. Clin. Biol.* **1992**, *16*, 92–95.
85. Larrey, D.; Vial, T.; Pauwels, A.; Castot, A.; Biour, M.; David, M.; Michel, H. Hepatitis after germander (teucrium chamaedrys) administration: Another instance of herbal medicine hepatotoxicity. *Ann. Intern. Med.* **1992**, *117*, 129–132.
86. Ben Yahia, M.; Mavier, P.; Metreau, J.M.; Zafrani, E.S.; Fabre, M.; Gatineau-Saillant, G.; Dhumeaux, D.; Mallat, A. Chronic active hepatitis and cirrhosis induced by wild germander. 3 cases. *Gastroenterol. Clin. Biol.* **1993**, *17*, 959–962.
87. Laliberte, L.; Villeneuve, J.P. Hepatitis after the use of germander, a herbal remedy. *CMAJ* **1996**, *154*, 1689–1692.
88. Mattei, A.; Rucay, P.; Samuel, D.; Feray, C.; Reynes, M.; Bismuth, H. Liver transplantation for severe acute liver failure after herbal medicine (teucrium polium) administration. *J. Hepatol.* **1995**, *22*, 597.
89. Smith, A.; Dillon, J. Acute liver injury associated with the use of herbal preparations containing glucosamine: Three case studies. *BMJ Case Rep.* **2009**, *2009*.
90. Ossendza, R.A.; Grandval, P.; Chinoune, F.; Rocher, F.; Chapel, F.; Bernardini, D. acute cholestatic hepatitis due to glucosamine forte. *Gastroenterol. Clin. Biol.* **2007**, *31*, 449–450.
91. Cerda, C.; Bruguera, M.; Pares, A. Hepatotoxicity associated with glucosamine and chondroitin sulfate in patients with chronic liver disease. *World J. Gastroenterol.* **2013**, *19*, 5381–5384.
92. Im, S.G.; Yoo, S.H.; Jeon, D.O.; Cho, H.J.; Choi, J.Y.; Paik, S.; Park, Y.M. Chelidonium majus-induced acute hepatitis. *Ewha Med J* **2014**, *37*, 60–63.
93. Stickel, F.; Poschl, G.; Seitz, H.K.; Waldherr, R.; Hahn, E.G.; Schuppan, D. Acute hepatitis induced by greater celandine (chelidonium majus). *Scand. J. Gastroenterol.* **2003**, *38*, 565–568.
94. Moro, P.A.; Cassetti, F.; Giugliano, G.; Falce, M.T.; Mazzanti, G.; Menniti-Ippolito, F.; Raschetti, R.; Santuccio, C. Hepatitis from greater celandine (chelidonium majus l.): Review of literature and report of a new case. *J. Ethnopharmacol.* **2009**, *124*, 328–332.
95. Pinto Garcia, V.; Vicente, P.R.; Barez, A.; Soto, I.; Candas, M.A.; Coma, A. hemolytic anemia induced by chelidonium majus. Clinical case. *Sangre (Barc)* **1990**, *35*, 401–403.
96. Benninger, J.; Schneider, H.T.; Schuppan, D.; Kirchner, T.; Hahn, E.G. Acute hepatitis induced by greater celandine (chelidonium majus). *Gastroenterology* **1999**, *117*, 1234–1237.
97. Crijns, A.P.; de Smet, P.A.; van den Heuvel, M.; Schot, B.W.; Haagsma, E.B. acute hepatitis after use of a herbal preparation with greater celandine (chelidonium majus). *Ned. Tijdschr. Geneeskd.* **2002**, *146*, 124–128.
98. Rifai, K.; Flemming, P.; Manns, M.P.; Trautwein, C. severe drug hepatitis caused by chelidonium. *Internist (Berl)* **2006**, *47*, 749–751.
99. Hardeman, E.; Van Overbeke, L.; Ilegems, S.; Ferrante, M. Acute hepatitis induced by greater celandine (chelidonium majus). *Acta Gastroenterol. Belg.* **2008**, *71*, 281–282.
100. Bjornsson, E.; Olsson, R. Serious adverse liver reactions associated with herbal weight-loss supplements. *J. Hepatol.* **2007**, *47*, 295–297; author reply 297-298.
101. Pillukat, M.H.; Bester, C.; Hensel, A.; Lechtenberg, M.; Petereit, F.; Beckebaum, S.; Muller, K.M.; Schmidt, H.H. Concentrated green tea extract induces severe acute hepatitis in a 63-year-old woman--a case report with pharmaceutical analysis. *J. Ethnopharmacol.* **2014**, *155*, 165–170.
102. Bonkovsky, H.L. Hepatotoxicity associated with supplements containing chinese green tea (camellia sinensis). *Ann. Intern. Med.* **2006**, *144*, 68–71.
103. Jimenez-Saenz, M.; Martinez-Sanchez Mdel, C. Acute hepatitis associated with the use of green tea infusions. *J. Hepatol.* **2006**, *44*, 616–617.
104. Molinari, M.; Watt, K.D.; Kruszyna, T.; Nelson, R.; Walsh, M.; Huang, W.Y.; Nashan, B.; Peltekian, K. Acute liver failure induced by green tea extracts: Case report and review of the literature. *Liver Transpl.* **2006**, *12*, 1892–1895.
105. Patel, S.S.; Beer, S.; Kearney, D.L.; Phillips, G.; Carter, B.A. Green tea extract: A potential cause of acute liver failure. *World J. Gastroenterol.* **2013**, *19*, 5174–5177.
106. Gloro, R.; Hourmand-Ollivier, I.; Mosquet, B.; Mosquet, L.; Rousselot, P.; Salame, E.; Piquet, M.A.; Dao, T. Fulminant hepatitis during self-medication with hydroalcoholic extract of green tea. *Eur J Gastroenterol Hepatol* **2005**, *17*, 1135–1137.
107. Javaid, A.; Bonkovsky, H.L. Hepatotoxicity due to extracts of chinese green tea (camellia sinensis): A growing concern. *J. Hepatol.* **2006**, *45*, 334–335.
108. Bergman, J.; Schjott, J. Hepatitis caused by lotus-f3? *Basic Clin. Pharmacol. Toxicol.* **2009**, *104*, 414–416.
109. Yellapu, R.K.; Mittal, V.; Grewal, P.; Fiel, M.; Schiano, T. Acute liver failure caused by 'fat burners' and dietary supplements: A case report and literature review. *Can. J. Gastroenterol.* **2011**, *25*, 157–160.
110. Gao, H.; Li, N.; Wang, J.Y.; Zhang, S.C.; Lin, G. Definitive diagnosis of hepatic sinusoidal obstruction syndrome induced by pyrrolizidine alkaloids. *J. Dig. Dis.* **2012**, *13*, 33–39.
111. Lin, G.; Wang, J.Y.; Li, N.; Li, M.; Gao, H.; Ji, Y.; Zhang, F.; Wang, H.; Zhou, Y.; Ye, Y.; et al*.* Hepatic sinusoidal obstruction syndrome associated with consumption of gynura segetum. *J Hepatol* **2011**, *54*, 666–673.
112. Dai, H.F.; Gao, Y.; Yang, M.; Yu, C.H.; Gu, Z.Y.; Chen, W.X. Hepatic veno-occlusive disease induced by gymura segetum: Report of two cases. *Hepatobiliary Pancreat Dis. Int.* **2006**, *5*, 406–408.
113. Chen, M.Y.; Cai, J.T.; Du, Q. Hepatic veno-occlusive disease associated with the use of gynura segetum. *Eur. J. Intern. Med.* **2007**, *18*, 609.
114. Kim, Y.J.; Ryu, S.L.; Shim, J.W.; Kim, D.S.; Shim, J.Y.; Park, M.S.; Jung, H.L. A pediatric case of toxic hepatitis induced by hovenia dulcis. *Pediatric Gastroenterol. Hepatol. Nutr.* **2012**, *15*, 111–116.
115. Hainer, M.I.; Tsai, N.; Komura, S.T.; Chiu, C.L. Fatal hepatorenal failure associated with hydrazine sulfate. *Ann. Intern. Med.* **2000**, *133*, 877–880.
116. Steenkamp, V.; Stewart, M.J.; Zuckerman, M. Detection of poisoning by impila (callilepis laureola) in a mother and child. *Hum. Exp. Toxicol.* **1999**, *18*, 594–597.
117. Daram, S.R.; Hayashi, P.H. Acute liver failure due to iron overdose in an adult. *South Med. J.* **2005**, *98*, 241–244.
118. Woolf, G.M.; Petrovic, L.M.; Rojter, S.E.; Wainwright, S.; Villamil, F.G.; Katkov, W.N.; Michieletti, P.; Wanless, I.R.; Stermitz, F.R.; Beck, J.J.; et al*.* Acute hepatitis associated with the chinese herbal product jin bu huan. *Ann. Intern. Med.* **1994**, *121*, 729–735.
119. Horowitz, R.S.; Feldhaus, K.; Dart, R.C.; Stermitz, F.R.; Beck, J.J. The clinical spectrum of jin bu huan toxicity. *Arch. Intern. Med.* **1996**, *156*, 899–903.
120. Picciotto, A.; Campo, N.; Brizzolara, R.; Giusto, R.; Guido, G.; Sinelli, N.; Lapertosa, G.; Celle, G. Chronic hepatitis induced by jin bu huan. *J. Hepatol.* **1998**, *28*, 165–167.
121. Centers for Disease, C.; Prevention. Jin bu huan toxicity in adults--los angeles, 1993. *Morb. Mortal. Wkly. Rep.* **1993**, *42*, 920–922.
122. Divinsky, M. Case report: Jin bu huan--not so benign herbal medicine. *Can. Fam. Physician.* **2002**, *48*, 1640–1642.
123. Koruk, S.T.; Ozyilkan, E.; Kaya, P.; Colak, D.; Donderici, O.; Cesaretli, Y. Juniper tar poisoning. *Clin. Toxicol. (Phila)* **2005**, *43*, 47–49.
124. Achour, S.; Abourazzak, S.; Mokhtari, A.; Soulaymani, A.; Soulaymani, R.; Hida, M. Juniper tar (cade oil) poisoning in new born after a cutaneous application. *BMJ Case Rep.* **2011**, *2011*.
125. Stickel, F.; Baumuller, H.M.; Seitz, K.; Vasilakis, D.; Seitz, G.; Seitz, H.K.; Schuppan, D. Hepatitis induced by kava (piper methysticum rhizoma). *J. Hepatol.* **2003**, *39*, 62–67.
126. Christl, S.U.; Seifert, A.; Seeler, D. Toxic hepatitis after consumption of traditional kava preparation. *J. Travel Med.* **2009**, *16*, 55–56.
127. Gow, P.J.; Connelly, N.J.; Hill, R.L.; Crowley, P.; Angus, P.W. Fatal fulminant hepatic failure induced by a natural therapy containing kava. *Med. J. Aust.* **2003**, *178*, 442–443.
128. Strahl, S.; Ehret, V.; Dahm, H.H.; Maier, K.P. necrotizing hepatitis after taking herbal remedies. *Dtsch. Med. Wochenschr.* **1998**, *123*, 1410–1414.
129. Russmann, S.; Lauterburg, B.H.; Helbling, A. Kava hepatotoxicity. *Ann. Intern. Med.* **2001**, *135*, 68–69.
130. Brauer, R.B.; Stangl, M.; Stewart, J.R.; Pfab, R.; Becker, K. Acute liver failure after administration of herbal tranquilizer kava-kava (piper methysticum). *J. Clin. Psychiatry* **2003**, *64*, 216–218.
131. Escher, M.; Desmeules, J.; Giostra, E.; Mentha, G. Hepatitis associated with kava, a herbal remedy for anxiety. *BMJ* **2001**, *322*, 139.
132. Bujanda, L.; Palacios, A.; Silvarino, R.; Sanchez, A.; Munoz, C. kava-induced acute icteric hepatitis. *Gastroenterol. Hepatol.* **2002**, *25*, 434–435.
133. Centers for Disease, C.; Prevention. Hepatic toxicity possibly associated with kava-containing products--united states, germany, and switzerland, 1999-2002. *Morb. Mortal. Wkly. Rep.* **2002**, *51*, 1065–1067.
134. Alhaddad, O.M.; Elsabaawy, M.M.; Rewisha, E.A.; Salman, T.A.; Kohla, M.A.; Ehsan, N.A.; Waked, I.A. Khat-induced liver injuries: A report of two cases. *Arab. J. Gastroenterol.* **2016**, *17*, 45–48.
135. Roelandt, P.; George, C.; d'Heygere, F.; Aerts, R.; Monbaliu, D.; Laleman, W.; Cassiman, D.; Verslype, C.; van Steenbergen, W.; Pirenne, J.; et al*.* Acute liver failure secondary to khat (catha edulis)-induced necrotic hepatitis requiring liver transplantation: Case report. *Transplant Proc* **2011**, *43*, 3493–3495.
136. Forbes, M.P.; Raj, A.S.; Martin, J.; Lampe, G.; Powell, E.E. Khat-associated hepatitis. *Med. J. Aust.* **2013**, *199*, 498–499.
137. Brostoff, J.M.; Plymen, C.; Birns, J. Khat--a novel cause of drug-induced hepatitis. *Eur. J. Intern. Med.* **2006**, *17*, 383.
138. Srinivasan, R.; Smolinske, S.; Greenbaum, D. Probable gastrointestinal toxicity of kombucha tea: Is this beverage healthy or harmful? *J. Gen. Intern. Med.* **1997**, *12*, 643–644.
139. Gedela, M.; Potu, K.C.; Gali, V.L.; Alyamany, K.; Jha, L.K. A case of hepatotoxicity related to kombucha tea consumption. *S D Med.* **2016**, *69*, 26–28.
140. Sullivan, S. Acute cholestatic hepatitis due to kratom.
141. Dorman, C.; Wong, M.; Khan, A. Cholestatic hepatitis from prolonged kratom use: A case report. *Hepatology* **2015**, *61*, 1086–1087.
142. Kapp, F.G.; Maurer, H.H.; Auwarter, V.; Winkelmann, M.; Hermanns-Clausen, M. Intrahepatic cholestasis following abuse of powdered kratom (mitragyna speciosa). *J. Med. Toxicol.* **2011**, *7*, 227–231.
143. Yilmaz, B.; Yilmaz, B.; Aktas, B.; Unlu, O.; Roach, E.C. Lesser celandine (pilewort) induced acute toxic liver injury: The first case report worldwide. *World J Hepatol.* **2015**, *7*, 285–288.
144. Yuen, M.F.; Tam, S.; Fung, J.; Wong, D.K.; Wong, B.C.; Lai, C.L. Traditional chinese medicine causing hepatotoxicity in patients with chronic hepatitis b infection: A 1-year prospective study. *Aliment Pharmacol. Ther.* **2006**, *24*, 1179–1186.
145. Kumana, C.R.; Ng, M.; Lin, H.J.; Ko, W.; Wu, P.C.; Todd, D. Herbal tea induced hepatic veno-occlusive disease: Quantification of toxic alkaloid exposure in adults. *Gut* **1985**, *26*, 101–104.
146. Sinniah, D.; Baskaran, G. Margosa oil poisoning as a cause of reye's syndrome. *Lancet* **1981**, *1*, 487–489.
147. Sinniah, D.; Baskaran, G.; Looi, L.M.; Leong, K.L. Reye-like syndrome due to margosa oil poisoning: Report of a case with postmortem findings. *Am. J. Gastroenterol.* **1982**, *77*, 158–161.
148. Senanayake, M.P.; Rupasinghe, S.; Dissanayake, P.V. Margosa (kohomba) oil induced toxic encephalopathy following home remedy for intestinal worms. *Ceylon. Med. J.* **2009**, *54*, 140.
149. Stadlbauer, V.; Fickert, P.; Lackner, C.; Schmerlaib, J.; Krisper, P.; Trauner, M.; Stauber, R.E. Hepatotoxicity of noni juice: Report of two cases. *World J Gastroenterol* **2005**, *11*, 4758–4760.
150. Yuce, B.; Gulberg, V.; Diebold, J.; Gerbes, A.L. Hepatitis induced by noni juice from morinda citrifolia: A rare cause of hepatotoxicity or the tip of the iceberg? *Digestion* **2006**, *73*, 167–170.
151. Lopez-Cepero Andrada, J.M.; Lerma Castilla, S.; Fernandez Olvera, M.D.; Amaya Vidal, A. hepatotoxicity caused by a noni (morinda citrifolia) preparation. *Rev. Esp. Enferm. Dig.* **2007**, *99*, 179–181.
152. Stadlbauer, V.; Weiss, S.; Payer, F.; Stauber, R.E. Herbal does not at all mean innocuous: The sixth case of hepatotoxicity associated with morinda citrifolia (noni). *Am. J. Gastroenterol.* **2008**, *103*, 2406–2407.
153. Yu, E.L.; Sivagnanam, M.; Ellis, L.; Huang, J.S. Acute hepatotoxicity after ingestion of morinda citrifolia (noni berry) juice in a 14-year-old boy. *J Pediatr Gastroenterol Nutr* **2011**, *52*, 222–224.
154. Mrzljak, A.; Kosuta, I.; Skrtic, A.; Kanizaj, T.F.; Vrhovac, R. Drug-induced liver injury associated with noni (morinda citrifolia) juice and phenobarbital. *Case Rep. Gastroenterol.* **2013**, *7*, 19–24.
155. Millonig, G.; Stadlmann, S.; Vogel, W. Herbal hepatotoxicity: Acute hepatitis caused by a noni preparation (morinda citrifolia). *Eur. J. Gastroenterol. Hepatol.* **2005**, *17*, 445–447.
156. Teschke, R.; Frenzel, C.; Schulze, J.; Eickhoff, A. Spontaneous reports of primarily suspected herbal hepatotoxicity by pelargonium sidoides: Was causality adequately ascertained? *Regul. Toxicol. Pharmacol.* **2012**, *63*, 1–9.
157. Sullivan, J.B., Jr.; Rumack, B.H.; Thomas, H., Jr.; Peterson, R.G.; Bryson, P. Pennyroyal oil poisoning and hepatotoxicity. *JAMA* **1979**, *242*, 2873–2874.
158. Bae, S.H.; Kim, D.H.; Bae, Y.S.; Lee, K.J.; Kim, D.W.; Yoon, J.B.; Hong, J.H.; Kim, S.H. toxic hepatitis associated with polygoni multiflori. *Korean J. Hepatol.* **2010**, *16*, 182–186.
159. Jung, K.A.; Min, H.J.; Yoo, S.S.; Kim, H.J.; Choi, S.N.; Ha, C.Y.; Kim, H.J.; Kim, T.H.; Jung, W.T.; Lee, O.J.; et al. Drug-induced liver injury: Twenty five cases of acute hepatitis following ingestion of polygonum multiflorum thunb. *Gut Liver* **2011**, *5*, 493–499.
160. Dong, H.; Slain, D.; Cheng, J.; Ma, W.; Liang, W. Eighteen cases of liver injury following ingestion of polygonum multiflorum. *Complement. Ther. Med.* **2014**, *22*, 70–74.
161. Cardenas, A.; Restrepo, J.C.; Sierra, F.; Correa, G. Acute hepatitis due to shen-min: A herbal product derived from polygonum multiflorum. *J. Clin. Gastroenterol.* **2006**, *40*, 629–632.
162. Cheung, W.I.; Tse, M.L.; Ngan, T.; Lin, J.; Lee, W.K.; Poon, W.T.; Mak, T.W.; Leung, V.K.; Chau, T.N. Liver injury associated with the use of fructus psoraleae (bol-gol-zhee or bu-gu-zhi) and its related proprietary medicine. *Clin. Toxicol. (Phila)* **2009**, *47*, 683–685.
163. Nam, S.W.; Baek, J.T.; Lee, D.S.; Kang, S.B.; Ahn, B.M.; Chung, K.W. A case of acute cholestatic hepatitis associated with the seeds of psoralea corylifolia (boh-gol-zhee). *Clin. Toxicol. (Phila)* **2005**, *43*, 589–591.
164. Markin, R.S.; Donovan, J.P.; Shaw, B.W., Jr.; Zetterman, R.K. Fulminant hepatic failure after methotrexate and puva therapy for psoriasis. *J. Clin. Gastroenterol.* **1993**, *17*, 311–313.
165. Stephens, R.B.; Cooper, A. Hepatitis from 5-methoxypsoralen occurring in a patient with previous flucloxacillin hepatitis. *Australas J. Dermatol.* **1999**, *40*, 217–219.
166. Smith, D.A.; MacDonald, S. A rare case of acute hepatitis induced by use of babchi seeds as an ayurvedic remedy for vitiligo. *BMJ Case Rep.* **2014**, *2014*.
167. Mazzanti, G.; Moro, P.A.; Raschi, E.; Da Cas, R.; Menniti-Ippolito, F. Adverse reactions to dietary supplements containing red yeast rice: Assessment of cases from the italian surveillance system. *Br. J. Clin. Pharmacol.* **2017**, *83*, 894–908.
168. Roselle, H.; Ekatan, A.; Tzeng, J.; Sapienza, M.; Kocher, J. Symptomatic hepatitis associated with the use of herbal red yeast rice. *Ann. Intern. Med.* **2008**, *149*, 516–517.
169. Grieco, A.; Miele, L.; Pompili, M.; Biolato, M.; Vecchio, F.M.; Grattagliano, I.; Gasbarrini, G. Acute hepatitis caused by a natural lipid-lowering product: When "alternative" medicine is no "alternative" at all. *J. Hepatol.* **2009**, *50*, 1273–1277.
170. Yuen, M.F.; Ip, P.; Ng, W.K.; Lai, C.L. Hepatotoxicity due to a formulation of ganoderma lucidum (lingzhi). *J. Hepatol.* **2004**, *41*, 686–687.
171. Wanmuang, H.; Leopairut, J.; Kositchaiwat, C.; Wananukul, W.; Bunyaratvej, S. Fatal fulminant hepatitis associated with ganoderma lucidum (lingzhi) mushroom powder. *J. Med. Assoc. Thai* **2007**, *90*, 179–181.
172. Lapi, F.; Gallo, E.; Giocaliere, E.; Vietri, M.; Baronti, R.; Pieraccini, G.; Tafi, A.; Menniti-Ippolito, F.; Mugelli, A.; Firenzuoli, F.; et al. Acute liver damage due to serenoa repens: A case report. *Br. J. Clin. Pharmacol.* **2010**, *69*, 558–560.
173. Hamid, S.; Rojter, S.; Vierling, J. Protracted cholestatic hepatitis after the use of prostata. *Ann. Intern. Med* **1997**, *127*, 169–170.
174. Vanderperren, B.; Rizzo, M.; Angenot, L.; Haufroid, V.; Jadoul, M.; Hantson, P. Acute liver failure with renal impairment related to the abuse of senna anthraquinone glycosides. *Ann. Pharmacother.* **2005**, *39*, 1353–1357.
175. Woolf, G. In *Senna-induced hepatotoxicity*, Hepatology, 1999; Co, W.B.S., INDEPENDENCE SQUARE WEST CURTIS CENTER, STE 300, PHILADELPHIA, PA 19106-3399 USA: pp 550A-550A.
176. Beuers, U.; Spengler, U.; Pape, G.R. Hepatitis after chronic abuse of senna. *Lancet* **1991**, *337*, 372–373.
177. Seybold, U.; Landauer, N.; Hillebrand, S.; Goebel, F.D. Senna-induced hepatitis in a poor metabolizer. *Ann. Intern. Med.* **2004**, *141*, 650–651.
178. Sonmez, A.; Yilmaz, M.I.; Mas, R.; Ozcan, A.; Celasun, B.; Dogru, T.; Taslipinar, A.; Kocar, I.H. Subacute cholestatic hepatitis likely related to the use of senna for chronic constipation. *Acta Gastroenterol.. Belg.* **2005**, *68*, 385–387.
179. Soyuncu, S.; Cete, Y.; Nokay, A.E. Portal vein thrombosis related to cassia angustifolia. *Clin. Toxicol. (Phila)* **2008**, *46*, 774–777.
180. Sohn, C.H.; Cha, M.I.; Oh, B.J.; Yeo, W.H.; Lee, J.H.; Kim, W.; Lim, K.S. Liver transplantation for acute toxic hepatitis due to herbal medicines and preparations. *J. Korean Soc.Clin. Toxicol.***2008**, *6*, 110–116.
181. Linnebur, S.A.; Rapacchietta, O.C.; Vejar, M. Hepatotoxicity associated with chinese skullcap contained in move free advanced dietary supplement: Two case reports and review of the literature. *Pharmacotherapy* **2010**, *30*, 750, 258e–262e.
182. Dhanasekaran, R.; Owens, V.; Sanchez, W. Chinese skullcap in move free arthritis supplement causes drug induced liver injury and pulmonary infiltrates. *Case Rep. Hepatol.* **2013**, *2013*, 965092.
183. Papafragkakis, C.; Ona, M.A.; Reddy, M.; Anand, S. Acute hepatitis after ingestion of a preparation of chinese skullcap and black catechu for joint pain. *Case Rep.Hepatol.* **2016**, *2016*, 4356749.
184. Yang, L.; Aronsohn, A.; Hart, J.; Jensen, D. Herbal hepatoxicity from chinese skullcap: A case report. *World J. Hepatol.* **2012**, *4*, 231–233.
185. MacGregor, F.B.; Abernethy, V.E.; Dahabra, S.; Cobden, I.; Hayes, P.C. Hepatotoxicity of herbal remedies. *BMJ* **1989**, *299*, 1156–1157.
186. Caldwell, S.H.; Feeley, J.W.; Wieboldt, T.F.; Featherston, P.L.; Dickson, R.C. Acute hepatitis with use of over-the-counter herbal remedies. *Va. Med. Q.* **1994**, *121*, 31–33.
187. Hullar, T.E.; Sapers, B.L.; Ridker, P.M.; Jenkins, R.L.; Huth, T.S.; Farraye, F.A. Herbal toxicity and fatal hepatic failure. *Am J Med* **1999**, *106*, 267–268.
188. Farre, M.; Xirgu, J.; Salgado, A.; Peracaula, R.; Reig, R.; Sanz, P. Fatal oxalic acid poisoning from sorrel soup. *Lancet* **1989**, *2*, 1524.
189. Dominguez Jimenez, J.L.; Pleguezuelo Navarro, M.; Guiote Malpartida, S.; Fraga Rivas, E.; Montero Alvarez, J.L.; Poyato Gonzalez, A. hepatotoxicity associated with hypericum (st. John's wort). *Gastroenterol. Hepatol.* **2007**, *30*, 54–55.
190. Etogo-Asse, F.; Boemer, F.; Sempoux, C.; Geubel, A. Acute hepatitis with prolonged cholestasis and disappearance of interlobular bile ducts following tibolone and hypericum perforatum (st. John's wort). Case of drug interaction? *Acta. Gastroenterol. Belg.* **2008**, *71*, 36–38.
191. Hsu, L.M.; Huang, Y.S.; Chang, F.Y.; Lee, S.D. 'Fat burner' herb, usnic acid, induced acute hepatitis in a family. *J. Gastroenterol. Hepatol.* **2005**, *20*, 1138–1139.
192. Durazo, F.A.; Lassman, C.; Han, S.H.; Saab, S.; Lee, N.P.; Kawano, M.; Saggi, B.; Gordon, S.; Farmer, D.G.; Yersiz, H.; et al. Fulminant liver failure due to usnic acid for weight loss. *Am. J. Gastroenterol.* **2004**, *99*, 950–952.
193. Sanchez, W.; Maple, J.T.; Burgart, L.J.; Kamath, P.S. Severe hepatotoxicity associated with use of a dietary supplement containing usnic acid. *Mayo. Clin. Proc.* **2006**, *81*, 541–544.
194. Vassiliadis, T.; Anagnostis, P.; Patsiaoura, K.; Giouleme, O.; Katsinelos, P.; Mpoumponaris, A.; Eugenidis, N. Valeriana hepatotoxicity. *Sleep Med.* **2009**, *10*, 935.
195. Mennecier, D.; Saloum, T.; Dourthe, P.M.; Bronstein, J.A.; Thiolet, C.; Farret, O. acute hepatitis after phytotherapy. *Presse. Med.* **1999**, *28*, 966.
196. Cohen, D.L.; Del Toro, Y. A case of valerian-associated hepatotoxicity. *J. Clin. Gastroenterol.* **2008**, *42*, 961–962.
197. Kia, Y.H.; Alexander, S.; Dowling, D.; Standish, R. A case of steroid-responsive valerian-associated hepatitis. *Intern. Med. J.* **2016**, *46*, 118–119.
198. Russell, R.M.; Boyer, J.L.; Bagheri, S.A.; Hruban, Z. Hepatic injury from chronic hypervitaminosis a resulting in portal hypertension and ascites. *N. Engl. J. Med.* **1974**, *291*, 435–440.
199. Russell, R.M. The vitamin a spectrum: From deficiency to toxicity. *Am. J. Clin. Nutr.* **2000**, *71*, 878–884.
200. Harb, J.N.; Taylor, Z.A.; Khullar, V.; Sattari, M. Rare cause of acute hepatitis: A common energy drink. *BMJ Case Rep.* **2016**, *2016*.
201. Bassan, M. A case for immediate-release niacin. *Heart Lung* **2012**, *41*, 95–98.
202. Etchason, J.A.; Miller, T.D.; Squires, R.W.; Allison, T.G.; Gau, G.T.; Marttila, J.K.; Kottke, B.A. Niacin-induced hepatitis: A potential side effect with low-dose time-release niacin. *Mayo. Clin. Proc.* **1991**, *66*, 23–28.
