# Supplementary material for: The Development of a Database for Herbal and Dietary Supplement Induced Liver Toxicity
Source: Int J Mol Sci. 2018 Sep 28;19(10):2955. doi: 10.3390/ijms19102955 (PMC6213387; doi:10.3390/ijms19102955)
Supplement: Supplementary file 1 [file ijms-19-02955-s001.zip › ijms-344147-suplementary/ijms-344147-Table S2-proofreading.docx]

**Table S2.** Bioactivations of some HDS ingredients.

| **HDS ingredients** | **Herbal sources** | **Involved CYP enzymes** | **Bioactivation process** | **Reference** |
| --- | --- | --- | --- | --- |
| avalactones | Kava | 1A2,2C9,2C19,2D6,3A4 | Quinone formation | [1,2] |
| Teucrin A | Germander | 3A4 | Epoxiation to enedial | [3,4] |
| Pulegone | Pennyroyal oil | 1A2, 2B6, 3A4 | Epoxiation to enedial | [5,6] |
| Safrole | Camphor | 2A6,2C9,2D6,2E1 | Cleavage processes | [7] |
| Corynoline | Corydalis | 2C9, 3A4,2C19 | Form ortho-quinone derivatives | [8] |
| Methoxalen | Psoralen | 2A6, 2B1 | Mechanism-based inactivation of CYP2A6,2B1 | [9,10] |
| Aristolochic acids | Aristolochia | 1A1,1A2 | Nitro reduction and O-demethylation | [11,12] |
| Alkenylbenzenes | Dill | 1A2,2A6, 2C19, 2D6, 2E1 | 1'-hydroxyestragole, O-Demethylation | [13,14] |
| Coumarin | Tonka bean | 2A13 | 3,4-epoxidation | [15,16] |
| Quercetin | Red onion | Unknown | Quinone methides formation | [13] |
| Retronecine‐type pyrrolizidine alkaloids | Comfrey | 3A4 | Pyrrolic ester formation | [17–19] |
| Heliotridine‐type pyrrolizidine alkaloids | Comfrey | 3A4 | Pyrrolic ester formation | [18,20] |
| Otonecine‐type pyrrolizidine alkaloids | Comfrey | 3A4,3A5 | Pyrrolic ester formation | [18,19,21] |

**Reference**

1. Johnson, B.M.; Qiu, S.X.; Zhang, S.; Zhang, F.; Burdette, J.E.; Yu, L.; Bolton, J.L.; van Breemen, R.B. Identification of novel electrophilic metabolites of piper methysticum forst (kava). *Chem. Res. Toxicol.* **2003**, *16*, 733–740.
2. Mathews, J.M.; Etheridge, A.S.; Black, S.R. Inhibition of human cytochrome p450 activities by kava extract and kavalactones. *Drug Metab. Dispos.* **2002**, *30*, 1153–1157.
3. Druckova, A.; Marnett, L.J. Characterization of the amino acid adducts of the enedial derivative of teucrin a. *Chem. Res. .Toxicol.* **2006**, *19*, 1330–1340.
4. Lekehal, M.; Pessayre, D.; Lereau, J.M.; Moulis, C.; Fouraste, I.; Fau, D. Hepatotoxicity of the herbal medicine germander: Metabolic activation of its furano diterpenoids by cytochrome p450 3a depletes cytoskeleton-associated protein thiols and forms plasma membrane blebs in rat hepatocytes. *Hepatology* **1996**, *24*, 212–218.
5. Khojasteh-Bakht, S.C.; Chen, W.; Koenigs, L.L.; Peter, R.M.; Nelson, S.D. Metabolism of (r)-(+)-pulegone and (r)-(+)-menthofuran by human liver cytochrome p-450s: Evidence for formation of a furan epoxide. *Drug Metab. Dispos.* **1999**, *27*, 574–580.
6. Lassila, T.; Mattila, S.; Turpeinen, M.; Pelkonen, O.; Tolonen, A. Tandem mass spectrometric analysis of s- and n-linked glutathione conjugates of pulegone and menthofuran and identification of p450 enzymes mediating their formation. *Rapid Commun. Mass Spectrom.* **2016**, *30*, 917–926.
7. Yang, A.H.; Zhang, L.; Zhi, D.X.; Liu, W.L.; Gao, X.; He, X. Identification and analysis of the reactive metabolites related to the hepatotoxicity of safrole. *Xenobiotica* **2017**, 1–9.
8. Mao, X.; Peng, Y.; Zheng, J. In vitro and in vivo characterization of reactive intermediates of corynoline. *Drug Metab. Dispos.* **2015**, *43*, 1491–1498.
9. Fang, Z.Z.; Zhang, Y.Y.; Ge, G.B.; Liang, S.C.; Sun, D.X.; Zhu, L.L.; Dong, P.P.; Cao, Y.F.; Yang, L. Identification of cytochrome p450 (cyp) isoforms involved in the metabolism of corynoline, and assessment of its herb-drug interactions. *Phytother. Res.* **2011**, *25*, 256–263.
10. Labbe, G.; Descatoire, V.; Beaune, P.; Letteron, P.; Larrey, D.; Pessayre, D. Suicide inactivation of cytochrome p-450 by methoxsalen. Evidence for the covalent binding of a reactive intermediate to the protein moiety. *J. Pharmacol. Exp. Ther.* **1989**, *250*, 1034–1042.
11. Chan, W.; Cui, L.; Xu, G.; Cai, Z. Study of the phase i and phase ii metabolism of nephrotoxin aristolochic acid by liquid chromatography/tandem mass spectrometry. *Rapid Commun. Mass Spectrom.* **2006**, *20*, 1755–1760.
12. Chan, W.; Luo, H.B.; Zheng, Y.; Cheng, Y.K.; Cai, Z. Investigation of the metabolism and reductive activation of carcinogenic aristolochic acids in rats. *Drug Metab. Dispos.* **2007**, *35*, 866–874.
13. Rietjens, I.M.; Boersma, M.G.; van der Woude, H.; Jeurissen, S.M.; Schutte, M.E.; Alink, G.M. Flavonoids and alkenylbenzenes: Mechanisms of mutagenic action and carcinogenic risk. *Mutat. Res.* **2005**, *574*, 124–138.
14. Jeurissen, S.M.; Punt, A.; Boersma, M.G.; Bogaards, J.J.; Fiamegos, Y.C.; Schilter, B.; van Bladeren, P.J.; Cnubben, N.H.; Rietjens, I.M. Human cytochrome p450 enzyme specificity for the bioactivation of estragole and related alkenylbenzenes. *Chem. Res. Toxicol.* **2007**, *20*, 798–806.
15. Lake, B.G. Coumarin metabolism, toxicity and carcinogenicity: Relevance for human risk assessment. *Food Chem. Toxicol.* **1999**, *37*, 423–453.
16. von Weymarn, L.B.; Murphy, S.E. Cyp2a13-catalysed coumarin metabolism: Comparison with cyp2a5 and cyp2a6. *Xenobiotica* **2003**, *33*, 73–81.
17. Mei, N.; Guo, L.; Fu, P.P.; Fuscoe, J.C.; Luan, Y.; Chen, T. Metabolism, genotoxicity, and carcinogenicity of comfrey. *J. Toxicol. Environ. Health B Crit. Rev.* **2010**, *13*, 509–526.
18. Fu, P.P.; Xia, Q.; Lin, G.; Chou, M.W. Pyrrolizidine alkaloids--genotoxicity, metabolism enzymes, metabolic activation, and mechanisms. *Drug Metab. Rev.* **2004**, *36*, 1–55.
19. Ruan, J.; Yang, M.; Fu, P.; Ye, Y.; Lin, G. Metabolic activation of pyrrolizidine alkaloids: Insights into the structural and enzymatic basis. *Chem. Res. Toxicol.* **2014**, *27*, 1030–1039.
20. Fashe, M.M.; Juvonen, R.O.; Petsalo, A.; Rasanen, J.; Pasanen, M. Species-specific differences in the in vitro metabolism of lasiocarpine. *Chem. Res. Toxicol.* **2015**, *28*, 2034–2044.
21. Lin, G.; Cui, Y.Y.; Hawes, E.M. Characterization of rat liver microsomal metabolites of clivorine, an hepatotoxic otonecine-type pyrrolizidine alkaloid. *Drug Metab. Dispos.* **2000**, *28*, 1475–1483.
