# Supplementary material for: The Development of a Database for Herbal and Dietary Supplement Induced Liver Toxicity
Source: Int J Mol Sci. 2018 Sep 28;19(10):2955. doi: 10.3390/ijms19102955 (PMC6213387; doi:10.3390/ijms19102955)
Supplement: Supplementary file 1 [file ijms-19-02955-s001.zip › ijms-344147-suplementary/ijms-344147-Table S3-proofreading.docx]

**Table S3.** Selected mechanistic studies of HDS hepatotoxicity.

| **HDS** | **Toxic ingredients** | **Relevant hepatotoxic mechanisms** | **References** |
| --- | --- | --- | --- |
| Black cohosh | Whole product | Oxidative stress, mitochondrial toxicity | [1] |
|  | Ethanol extract | Mitochondrial toxicity, | [2] |
| Chaparral | Nordihydroguaiaretic acid | Oxidative stress, mitochondrial toxicity, ER stress | [3–6] |
| Germander | Diterpenoids | Bioactivation and GSH depletion | [7,8] |
| Greater celandine | Ethanol extract | Oxidative stress | [9] |
| Green tea extract | Epigallocatechin-3-gallate | Oxidative stress, mitochondrial toxicity | [10–14] |
| Kava | Pipermethystine | Mitochondrial toxicity | [15] |
|  | Flavokawain B | Oxidative stress | [16] |
| Neem oil | Whole product | Mitochondrial toxicity | [17,18] |
| Lipokinetix | Usnic acid | Mitochondrial toxicity, oxidative stress | [19] |
|  | Usnic acid | Autophagy | [20] |

**References**

1. Campos, L.B.; Gilglioni, E.H.; Garcia, R.F.; Brito Mdo, N.; Natali, M.R.; Ishii-Iwamoto, E.L.; Salgueiro-Pagadigorria, C.L. Cimicifuga racemosa impairs fatty acid beta-oxidation and induces oxidative stress in livers of ovariectomized rats with renovascular hypertension. *Free Radic. Biol. Med.* **2012**, *53*, 680–689.
2. Lude, S.; Torok, M.; Dieterle, S.; Knapp, A.C.; Kaeufeler, R.; Jaggi, R.; Spornitz, U.; Krahenbuhl, S. Hepatic effects of cimicifuga racemosa extract in vivo and in vitro. *Cell. Mol. Life Sci.* **2007**, *64*, 2848–2857.
3. Sahu, S.C.; Ruggles, D.I.; O'Donnell, M.W. Prooxidant activity and toxicity of nordihydroguaiaretic acid in clone-9 rat hepatocyte cultures. *Food Chem. Toxicol.* **2006**, *44*, 1751–1757.
4. Fujiwara, T.; Takami, N.; Misumi, Y.; Ikehara, Y. Nordihydroguaiaretic acid blocks protein transport in the secretory pathway causing redistribution of golgi proteins into the endoplasmic reticulum. *J. Biol. Chem.* **1998**, *273*, 3068–3075.
5. Fujiwara, T.; Misumi, Y.; Ikehara, Y. Dynamic recycling of ergic53 between the endoplasmic reticulum and the golgi complex is disrupted by nordihydroguaiaretic acid. *Biochem. Biophys. Res. Commun.* **1998**, *253*, 869–876.
6. Pardini, R.S.; Heidker, J.C.; Fletcher, D.C. Inhibition of mitochondrial electron transport by nor-dihydroguaiaretic acid (ndga). *Biochem Pharmacol* **1970**, *19*, 2695–2699.
7. Fau, D.; Lekehal, M.; Farrell, G.; Moreau, A.; Moulis, C.; Feldmann, G.; Haouzi, D.; Pessayre, D. Diterpenoids from germander, an herbal medicine, induce apoptosis in isolated rat hepatocytes. *Gastroenterology* **1997**, *113*, 1334–1346.
8. Lekehal, M.; Pessayre, D.; Lereau, J.M.; Moulis, C.; Fouraste, I.; Fau, D. Hepatotoxicity of the herbal medicine germander: Metabolic activation of its furano diterpenoids by cytochrome p450 3a depletes cytoskeleton-associated protein thiols and forms plasma membrane blebs in rat hepatocytes. *Hepatology* **1996**, *24*, 212–218.
9. Mazzanti, G.; Di Sotto, A.; Franchitto, A.; Mammola, C.L.; Mariani, P.; Mastrangelo, S.; Menniti-Ippolito, F.; Vitalone, A. Chelidonium majus is not hepatotoxic in wistar rats, in a 4 weeks feeding experiment. *J. Ethnopharmacol.* **2009**, *126*, 518–524.
10. Kucera, O.; Mezera, V.; Moravcova, A.; Endlicher, R.; Lotkova, H.; Drahota, Z.; Cervinkova, Z. In vitro toxicity of epigallocatechin gallate in rat liver mitochondria and hepatocytes. *Oxid. Med. Cell Longev.* **2015**, *2015*, 476180.
11. Wang, D.; Wang, Y.; Wan, X.; Yang, C.S.; Zhang, J. Green tea polyphenol (-)-epigallocatechin-3-gallate triggered hepatotoxicity in mice: Responses of major antioxidant enzymes and the nrf2 rescue pathway. *Toxicol Appl. Pharmacol.* **2015**, *283*, 65–74.
12. Galati, G.; Lin, A.; Sultan, A.M.; O'Brien, P.J. Cellular and in vivo hepatotoxicity caused by green tea phenolic acids and catechins. *Free Radic. Biol. Med.* **2006**, *40*, 570–580.
13. Lambert, J.D.; Kennett, M.J.; Sang, S.; Reuhl, K.R.; Ju, J.; Yang, C.S. Hepatotoxicity of high oral dose (-)-epigallocatechin-3-gallate in mice. *Food Chem. Toxicol.* **2010**, *48*, 409–416.
14. Elbling, L.; Weiss, R.M.; Teufelhofer, O.; Uhl, M.; Knasmueller, S.; Schulte-Hermann, R.; Berger, W.; Micksche, M. Green tea extract and (-)-epigallocatechin-3-gallate, the major tea catechin, exert oxidant but lack antioxidant activities. *FASEB J.* **2005**, *19*, 807–809.
15. Nerurkar, P.V.; Dragull, K.; Tang, C.S. In vitro toxicity of kava alkaloid, pipermethystine, in hepg2 cells compared to kavalactones. *Toxicol. Sci.* **2004**, *79*, 106–111.
16. Zhou, P.; Gross, S.; Liu, J.H.; Yu, B.Y.; Feng, L.L.; Nolta, J.; Sharma, V.; Piwnica-Worms, D.; Qiu, S.X. Flavokawain b, the hepatotoxic constituent from kava root, induces gsh-sensitive oxidative stress through modulation of ikk/nf-kappab and mapk signaling pathways. *FASEB J* **2010**, *24*, 4722–4732.
17. Koga, Y.; Yoshida, I.; Kimura, A.; Yoshino, M.; Yamashita, F.; Sinniah, D. Inhibition of mitochondrial functions by margosa oil: Possible implications in the pathogenesis of reye's syndrome. *Pediatr. Res.* **1987**, *22*, 184–187.
18. Trost, L.C.; Lemasters, J.J. The mitochondrial permeability transition: A new pathophysiological mechanism for reye's syndrome and toxic liver injury. *J. Pharmacol. Exp. Ther.* **1996**, *278*, 1000–1005.
19. Han, D.; Matsumaru, K.; Rettori, D.; Kaplowitz, N. Usnic acid-induced necrosis of cultured mouse hepatocytes: Inhibition of mitochondrial function and oxidative stress. *Biochem. Pharmacol.* **2004**, *67*, 439–451.
20. Chen, S.; Dobrovolsky, V.N.; Liu, F.; Wu, Y.; Zhang, Z.; Mei, N.; Guo, L. The role of autophagy in usnic acid-induced toxicity in hepatic cells. *Toxicol. Sci.* **2014**, *142*, 33–44.
